# Short-term and long-term effect of non-pharmacotherapy for adults with ADHD: a systematic review and network meta-analysis

**DOI:** 10.3389/fpsyt.2025.1516878

**Published:** 2025-01-31

**Authors:** Xinyue Yang, Lin Zhang, Jing Yu, Meng Wang

**Affiliations:** ^1^ College of Sports Science, Shenyang Normal University, Shenyang, China; ^2^ Department of Rehabilitation, West China Hospital Sichuan University Jintang Hospital, Chengdu, China

**Keywords:** adult ADHD, non-pharmacological therapy, network meta-analysis, CBT (cognitive behavioral therapy), mindfulness - ADHD - intervention - disorder - MBCT

## Abstract

**Background:**

Attention-Deficit/Hyperactivity Disorder (ADHD) is a lifelong neurodevelopmental condition with a global prevalence of 2.5% to 6.7% among adults. Non-pharmacological interventions have demonstrated effectiveness both as standalone treatments and adjuncts to pharmacotherapy in managing adult ADHD. Nevertheless, the comparative efficacy of these interventions, particularly with respect to diverse ADHD-related outcomes and their long-term impacts, remains insufficiently investigated.

**Objective:**

This study aims to evaluate and compare the short-term and long-term effects of various non-pharmacological therapies on core ADHD symptoms (inattention, hyperactivity, and impulsivity) and emotional disorders (depression and anxiety) in adults with ADHD and to rank these therapies accordingly.

**Methods:**

A systematic search was conducted for relevant randomized controlled trials (RCTs) in the Web of Science, PubMed, Cochrane Library, and EMBASE databases from inception to Sep 2024. Researchers independently screened and extracted data, and the analysis was performed using R version 4.3.2. Cochrane Risk of Bias tool version 2 (ROB2) and Confidence in Network Meta-Analysis (CINeMA) were used to assess the risk of bias and the certainty of the evidence. Standardized mean differences were estimated using network meta-analyses with random effects.

**Results:**

A total of 37 RCTs involving 2,289 participants and 10 non-pharmacological therapies were included. The risk of bias was classified as low in 24.3%, unclear in 27%, and high in 48.6%, while the CINeMA assessment indicated that confidence in the evidence was “very low” or “low” for most of the remaining treatments. Cognitive behavioral therapy (CBT) showed significantly greater effectiveness than the control group/condition in both the short-term (SMD: -4.43, 95%CI: -5.50 to -3.37) and long-term (SMD: -3.61, 95%CI: -4.66 to -2.56) core symptoms. Additionally, CBT shows both short-term and long-term efficacy for depression (SMD: -4.16, 95%CI: -5.51 to -2.81; SMD: -3.89, 95%CI: -5.95 to -1.83) and anxiety (SMD: -2.12, 95%CI: -3.18 to -1.07; SMD: -7.25, 95%CI: -10.57 to -3.94).

**Conclusion:**

CBT may be the most effective intervention for adults with ADHD and associated emotional disorders, while Mindfulness-based Cognitive Therapy (MC) is recommended as a preferable option for those without comorbidities. Caution is needed in interpreting our results, and high-quality RCTs are urgently required for more reliable insights.

**Systematic Review Registration:**

https://www.crd.york.ac.uk/prospero/display_record.php?, identifier CRD42024432912.

## Introduction

Attention-Deficit/Hyperactivity Disorder (ADHD) is a neurodevelopmental disorder typically diagnosed in childhood or adolescence, characterized by impairing levels of inattention, impulsivity, and hyperactivity, either individually or in combination. ADHD affects approximately 2.5% to 6.7% of adults globally (1–4). While historically considered a childhood-limited condition (5), ADHD is now understood to persist into adulthood, making it a lifelong disorder. In terms of core symptoms, inattention is more pronounced for adults with ADHD, along with a range of subtle symptoms, including boredom, distractibility, procrastination, restlessness and excessive mind-wondering (5, 6). Apart from core symptoms, ADHD also has a wide-ranging impact on adult patients, as they often experience impairments in executive function (7), metacognition (8), and emotional regulation (9), which contribute to maladaptive coping strategies, poor self-awareness, and the use of inappropriate emotional regulation techniques (e.g., avoidance) (10). These broad and complex impacts could significantly impair academic achievement (e.g., higher dropout rates and reduced opportunities for higher education), occupational performance (e.g., fewer employment opportunities and higher job turnover), social interactions, and emotional regulation, sleep problems and may even contribute to substance abuse and criminal behavior (10–15).

Although pharmacological treatments are considered first-line interventions for ADHD (16), they are often associated with adverse effects and suboptimal efficacy, leading to frequent discontinuation (17). The UK National Institute for Health and Care Excellence (NICE) therefore recommends combining non-pharmacological therapies with medication for adult ADHD management. A variety of nonpharmacological therapies (18–21), including Cognitive Behavioral Therapy (CBT), psychoeducation (PE), mindfulness, and physical exercise, have been explored for their ability to reduce ADHD core symptoms and related functional impairments. However, there is significant inconsistency and ongoing debate regarding the efficacy of these approaches. For instance, researchers (22, 23) suggest that exercise therapy effectively improves core ADHD symptoms and cognitive functions, while others (24) argue that methodological limitations in some existing meta-analyses may weaken the evidence, preventing exercise therapy from being recommended as a primary or secondary treatment for ADHD.

While numerous network meta-analyses (NMA) have sought to establish the efficacy and safety of different non-pharmacological interventions for ADHD (25–27), fewer studies have focused specifically on adults. A systematic review (28) of non-pharmacological interventions for adults indicates that these approaches may offer superior efficacy over control or inactive conditions in managing core behavioral symptoms, but no quantitative evidence has been provided. And a recent component NMA (29), comparing the effectiveness and safety of four treatment components: pharmacological therapies, psychological therapies, neurostimulatory therapy, and neurofeedback, suggested that non-pharmacological strategies showed inconsistent effects across raters (only effective when considering clinician-reported ratings), which aligns with the previous finding (30). However, further analysis is needed to assess the short- and long-term effects of these therapies, when used as standalone interventions (rather than as components of multimodal treatments), on improving core ADHD symptoms and emotional disorders, considering the potentially complex interactions between different effects.

To provide further insights, we conducted a systematic review and network meta-analysis of randomized controlled trials (RCTs) involving adults with ADHD to provide more reliable information on the efficacy of non-pharmacological therapies in managing core symptoms (including inattention, hyperactivity, and impulsivity) as well as emotional disorders (such as depression and anxiety). Furthermore, we examined the long-term effects of these non-pharmacological interventions, which are often compared with pharmacological treatments in the existing literature.

## Methods

Our study protocol has been registered in the International Prospective Register of Systematic Reviews (PROSPERO) with registration number CRD42024432912 and we followed the PRISMA extension guidelines for network meta-analyses.

### Search strategy

We searched PubMed, Web of Science, Cochrane Library (trials) and EMBASE from their inception to Sep 14, 2024, with no language restrictions. A combination of Medical Subject Headings (MeSH) terms and free-text terms were employed. Additionally, we screened the reference lists of identified reviews to find relevant RCTs that were not incorporated in the initial search. The detailed search strategy of PubMed is provided in **Supplementary Table 1**, and appropriate adjustments to this strategy were made for other databases (see **Supplementary Tables 2**-**4**).

### Selection criteria

We included RCTs (parallel-group or crossover designs) of at least three weeks’ duration, enrolling adults (aged ≥18 years) with a primary diagnosis of ADHD according to DSM-V (The Diagnostic and Statistical Manual of Mental Disorders, Fifth Edition), DSM-IV (The Diagnostic and Statistical Manual of Mental Disorders, Fourth Edition) or ADHS-SB (German ADHD self-rating scale for symptoms in adulthood) criteria. No restrictions were placed on ADHD subtype, sex, region, ethnicity, socioeconomic status, or comorbidities. Studies were included if they assessed any non-pharmacological therapy as monotherapy or in combination with usual treatments, as defined in their studies (such as continued psychopharmacology), compared with each other or with a waiting list, usual care, or an equivalent control intervention. We excluded studies that compared different types of the same therapy (e.g., SCP-NF vs. fNIRS-NF) or where the primary study outcomes were not reported. Full inclusion and exclusion criteria are detailed in the **Supplementary Table 5**.

### Procedures

Two independent researchers (YXY and ZL) conducted the article review and data extraction. We assessed the risk of bias and certainty of evidence using the Cochrane Risk of Bias tool version 2 (ROB2) (31) and Confidence in Network Meta-Analysis (CINeMA) (32) framework for network meta-analyses separately. Any discrepancies encountered during the process were resolved by consensus or by consulting a third party, including the corresponding author (WM and YJ).

### Outcomes

Our primary outcome measure is the change in the severity of ADHD core symptoms in both the short-term and long-term, as measured by clinician ratings, observer ratings, or self-assessment. If all three types of assessment were used in a study, clinician ratings were given preference. Although the rating scales varied, only validated scales (such as the ADHD Rating Scale and ADHD Self-Report Scale Symptom) were included. A full list of rating scales considered for inclusion is provided in **Supplementary Table 6**.

Secondary outcomes include changes in co-occurring depression and anxiety, primarily measured using the Beck Depression Inventory (BDI) and the Beck Anxiety Inventory (BAI), also considering both short-term and long-term effects.

### Statistical analysis

We performed network meta-analysis using the netmeta package in R within a frequentist framework to compare the effects of different non-pharmacological therapies (33). League tables were created for core ADHD symptoms, anxiety, and depression. The pooled effect size was expressed as SMD with a 95%CI. We used a random-effects model to synthesize the data. We applied the P-score, derived from the Surface Under the Cumulative Ranking Curve (34), to rank the efficacy of the interventions. The P-score ranges from 0 to 1, with higher scores indicating superior efficacy.

We assessed heterogeneity using multiple metrics, including the I² statistic, Cochran’s Q statistic, and τ² (tau-squared). The I² statistic quantifies the percentage of total variation across studies attributable to heterogeneity rather than chance, while Cochran’s Q statistic tests the null hypothesis of no heterogeneity. Additionally, τ² provides an estimate of between-study variance in effect sizes, collectively offering a comprehensive understanding of the degree of heterogeneity present in the network. To evaluate incoherence, we used node splitting to assess local incoherence (35) and design-by-treatment models (36) to assess global incoherence.

Regression analyses were conducted to explore potential sources of heterogeneity, including variables such as publication year, mean age, percentage of male participants, intervention duration, and frequency of intervention across studies. Considering the high heterogeneity in our study, we employed these analyses using the gemtc package in R which is considered more suitable for capturing and interpreting complex effect heterogeneity (37). To evaluate the robustness of our results, we reran the network meta-analysis using the centring values provided by the model. Subgroup analyses were conducted further to explore the specific impact of significant sources of heterogeneity. Comparison-corrected funnel plots were used to assess the risk of publication bias, and Egger’s test indicated publication bias when P < 0.05.

All statistical analyses were performed using R (version 4.3.2) software. Study quality was evaluated using the ROB2 tool, and the CINeMA framework was used to assess overall evidence certainty, including within-study bias, reporting bias, indirectness, imprecision, heterogeneity, and incoherence. The reasons and criteria for downgrading the level of evidence are provided after **Supplementary Table 18**.

## Results

### Study selection

**Figure 1:**
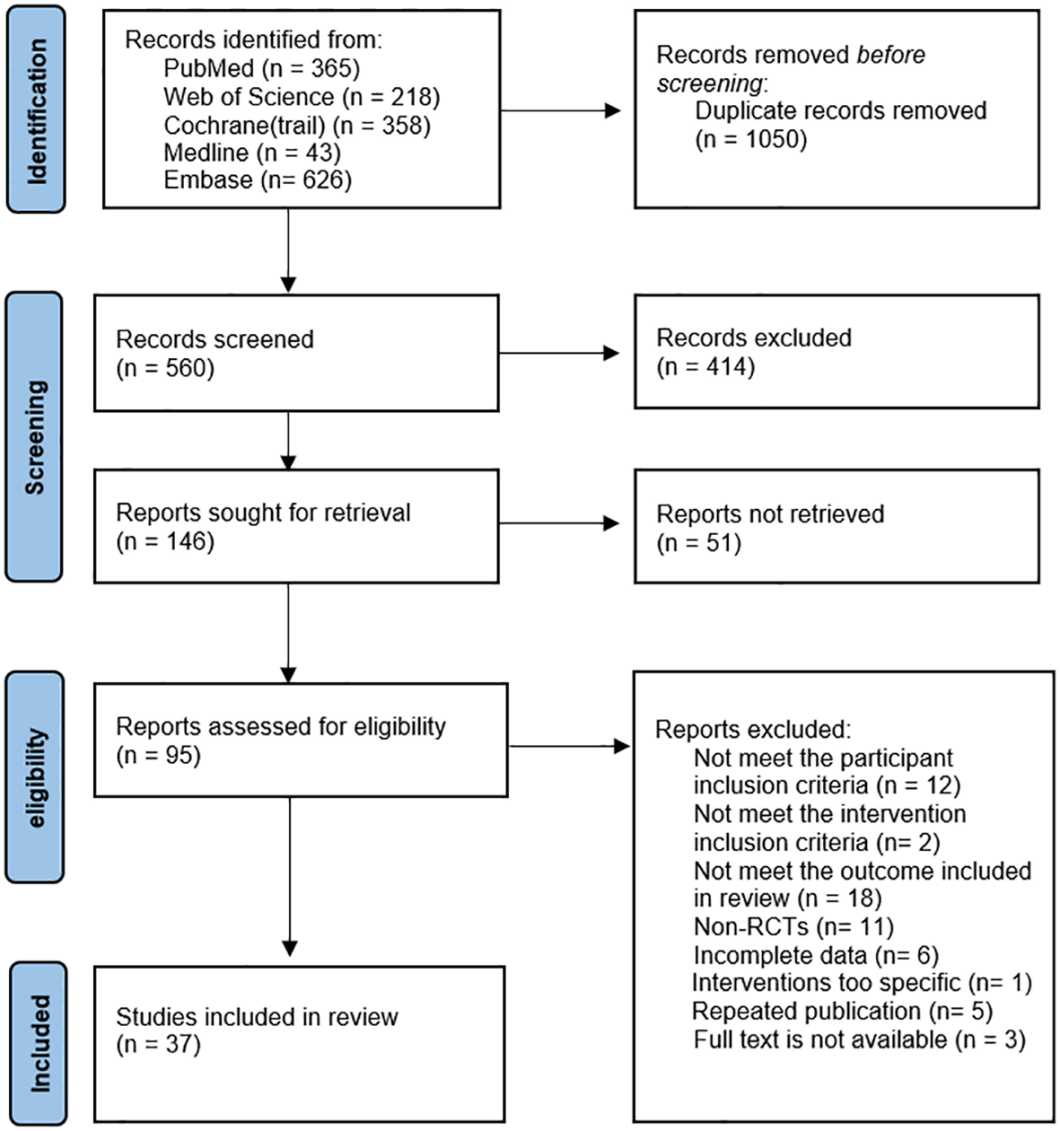
Selection of studies (PRISMA diagram).

The literature search, study selection, and data extraction were conducted between May 7, 2023, and April 5, 2024, with data analysis completed from April 6, 2024, to June 30, 2024. We updated the search results before submission and did not find any new studies that met the inclusion criteria. The study selection process is outlined in **Figure 1**.

A total of 10 non-pharmacological interventions were evaluated in our study, including CBT, Cognitive Therapy (CT), Mindfulness-based Cognitive Therapy (MC), Neurofeedback (NF), Noninvasive Brain Stimulation (NIBS), PE, Self-Alert Training (SAT), Tai Chi, Vitamin–Mineral Treatment (VMT), and Working Memory Training (WMT). The specific definitions of interventions can be found in the **Supplementary Table 7**. And 1610 citations were identified and screened based on predefined inclusion and exclusion criteria. A total of 37 studies were included in the network meta-analysis, comprising 2,289 adults with ADHD (1,006 males and 1,091 females; three studies did not report sex distribution). The main characteristics of these studies are detailed in the **Supplementary Table 8**.

### Primary outcomes

The primary outcomes of this study include the short-term and long-term efficacy in addressing ADHD core symptoms. For short-term efficacy, as assessed by clinicians, observers, or self-reports at the end of the intervention, CBT (SMD: -4.43, 95%CI: -5.50 to -3.37), CT (SMD: -4.02, 95%CI: -7.05 to -0.99), MC (SMD: -5.07, 95%CI: -7.29 to -2.84), NIBS (SMD: -2.38, 95%CI: -4.37 to -0.39) and PE (SMD: -6.38, 95%CI: -9.25 to -3.52 demonstrated significantly greater effectiveness compared to the control group/condition. While NF (SMD: -0.28, 95%CI: -2.47 to 1.91), SAT (SMD: -3.00, 95%CI: -7.22 to 1.22), Taichi (SMD: -2.20, 95%CI: -6.53 to 2.13), VMT (SMD: -3.02, 95%CI: -7.18 to 1.14), WMT (SMD: -1.37, 95%CI: -4.31 to 1.58) were not superior to control. Additionally, CBT (SMD: -4.15, 95%CI: -6.46 to -1.84) was significantly superior to NF, MC (SMD: -4.78, 95%CI: -7.89 to -1.68) was superior to NF, PE was superior to NF (SMD: 6.10, 95%CI: 2.53 to 9.67), NIBS (SMD: 4.10, 95%CI: 0.52 to 7.49) and WMT (SMD: -5.02, 95%CI: -9.13 to -0.91).

**Figure 2:**
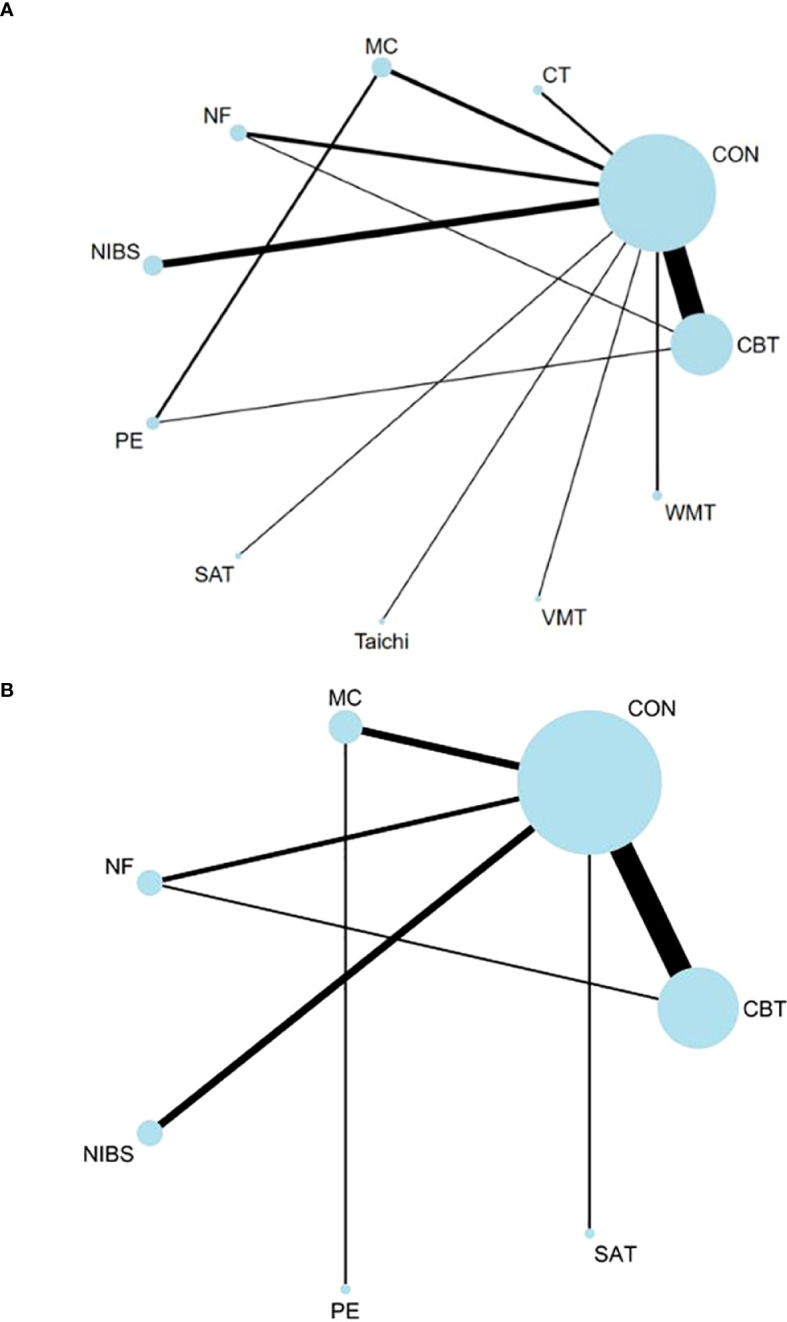
Network of ADHD core symptom. The width of the lines is proportional to the number of trials comparing each pair of treatments, and the size of each circle is proportional to the sample size. The number of trials for each treatment pair ranged from 17 (e.g. studies of short-term effects of CBT vs. Control) to 1 (in several comparisons). **(A)**The network of short-term effects on ADHD core symptoms; **(B)** The network of long-term effects on ADHD core symptoms. *ADHD*, Attention-deficit/hyperactivity disorder; *CBT*, Cognitive Behavioral Therapy; *CON*, Control; *CT*, Cognitive Therapy; *MC*, Mindfulness-based cognitive Therapy; *NF*, Neurofeedback; *NIBS*, Noninvasive Brain Stimulation; *PE*, Psychoeducation; *SAT*, Self-Alert Training; *Taichi*; *VMT,* Vitamin–mineral treatment; *WMT*, Working Memory Training;.

**Figure 3:**
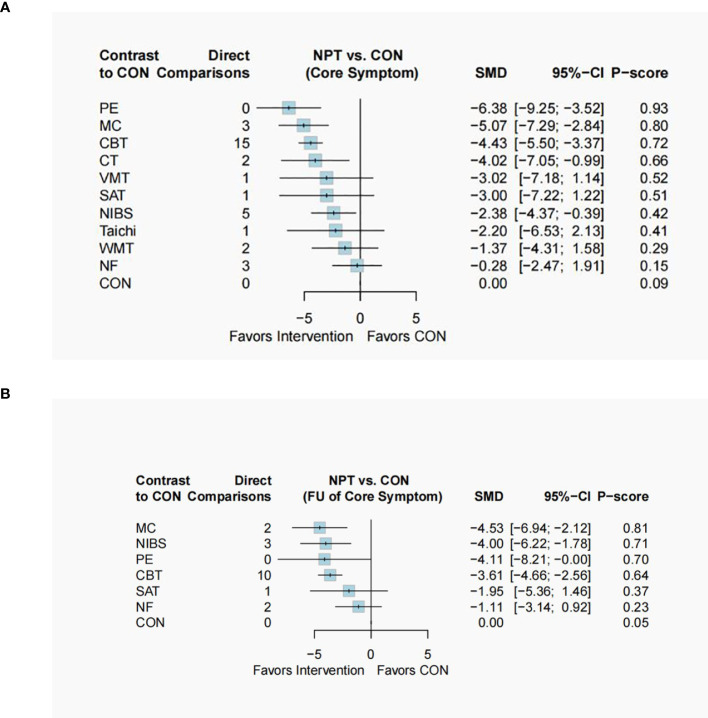
Forest plot of ADHD core symptom. The forest plot includes all eligible trials comparing efficacy against the control group (CON). **(A)**The forest plot of short-term effect on ADHD core symptoms; **(B)** The forest plot of long-term effect on ADHD core symptoms. *ADHD*, Attention-deficit and hyperactivity disorder; *CBT*, Cognitive Behavioral Therapy; *CT*, Cognitive Therapy; *MC*, Mindfulness-based cognitive Therapy; *NF*, Neurofeedback; *NIBS*, Noninvasive Brain Stimulation; *PE*, Psychoeducation; *SAT*, Self-Alert Training; *Taichi*; *VMT*, Vitamin–mineral treatment; *WMT*, Working Memory Training;.

Regarding the long-term effects of these interventions, CBT (SMD: -3.61, 95%CI: -4.66 to -2.56), MC (SMD: -4.53, 95%CI: -6.94 to -2.12), NIBS (SMD: -4.00, 95%CI: -6.22 to -1.78) and PE (SMD: -4.11, 95%CI: -8.21 to -0.00) remained significantly more effective than the control group. There were no available data for CT to determine whether the effect persists. The results of NF(SMD: -1.11, 95%CI: -3.14 to 0.92) and SAT (SMD: -1.95, 95%CI: -5.36 to 1.46) remain non-significant, consistent with their short-term effects. Furthermore, CBT (SMD: -2.50, 95%CI: -4.61 to -0.39) and MC (SMD: -3.42, 95%CI: -6.57 to -0.27) continued to be significantly more effective than NF. **Figure 2** provides the network plots for ADHD core symptoms. Results of the network meta-analyses for the primary outcomes are shown in **Figure 3**, **Tables 1**, **2**.

**Table 1:** League table of ADHD core symptoms.

Core Symptom	Comparison of treatments: Standardized Mean different(95%confidence intervals)/Effect of intervention in each row compared with intervention in each column									
**CBT**	NA	NA	**-6.16 (-10.42,-1.91)**	.NA	0.17 (-4.00, 4.33)	NA	NA	NA	NA	**-4.17 (-5.28,-3.06)**
-0.41 (-3.62, 2.80)	**CT**	NA	NA	NA	NA	NA	NA	NA	NA	**-4.02 (-7.05,-0.99)**
0.63 (-1.74, 3.01)	1.04 (-2.72, 4.80)	**MC**	NA	NA	2.21 (-0.74, 5.17)	NA	NA	NA	NA	**-5.69 (-8.14,-3.23)**
**-4.15 (-6.46,-1.84)**	-3.74 (-7.48, 0.00)	**-4.78 (-7.89,-1.68)**	**NF**	NA	NA	NA	NA	NA	NA	-0.99 (-3.52, 1.54)
-2.05 (-4.31, 0.20)	-1.64 (-5.27, 1.98)	-2.69 (-5.67, 0.29)	2.10 (-0.86, 5.05)	**NIBS**	NA	NA	NA	NA	NA	**-2.38 (-4.37,-0.39)**
1.95 (-0.93, 4.83)	2.36 (-1.81, 6.53)	1.32 (-1.22, 3.85)	**6.10 (2.53, 9.67)**	**4.01 (0.52, 7.49)**	**PE**	NA	NA	NA	NA	NA
-1.43 (-5.78, 2.92)	-1.02 (-6.22, 4.18)	-2.06 (-6.83, 2.71)	2.72 (-2.03, 7.47)	0.62 (-4.04, 5.29)	-3.38 (-8.48, 1.72)	**SAT**	NA	NA	NA	-3.00 (-7.22, 1.22)
-2.23 (-6.69, 2.23)	-1.82 (-7.11, 3.47)	-2.87 (-7.73, 2.00)	1.92 (-2.93, 6.77)	-0.18 (-4.94, 4.59)	-4.18 (-9.38, 1.01)	-0.80 (-6.85, 5.24)	**Taichi**	NA	NA	-2.20 (-6.53, 2.13)
-1.42 (-5.71, 2.88)	-1.00 (-6.15, 4.14)	-2.05 (-6.76, 2.67)	2.74 (-1.96, 7.44)	0.64 (-3.97, 5.25)	-3.37 (-8.42, 1.68)	0.02 (-5.91, 5.94)	0.82 (-5.19, 6.82)	**VMT**	NA	-3.02 (-7.18, 1.14)
-3.07 (-6.20, 0.07)	-2.65 (-6.88, 1.57)	**-3.70 (-7.39,-0.01)**	1.09 (-2.58, 4.76)	-1.01 (-4.56, 2.54)	**-5.02 (-9.13,-0.91)**	-1.63 (-6.78, 3.51)	-0.83 (-6.07, 4.40)	-1.65 (-6.75, 3.45)	**WMT**	-1.37 (-4.31, 1.58)
**-4.43 (-5.50,-3.37)**	**-4.02 (-7.05,-0.99)**	**-5.07 (-7.29,-2.84)**	-0.28 (-2.47, 1.91)	**-2.38 (-4.37,-0.39)**	**-6.38 (-9.25,-3.52)**	-3.00 (-7.22, 1.22)	-2.20 (-6.53, 2.13)	-3.02 (-7.18, 1.14)	-1.37 (-4.31, 1.58)	**CON**

Data are expressed as standardized mean differences (95%CI) between therapies. Significant results are highlighted in bold. Negative values favor the therapy in the row, and positive values favor the therapy in the column. Non-pharmacological therapies are listed in alphabetical order. The top section displays direct comparison results, while the bottom section shows results from mixed comparisons. *CBT*, Cognitive Behavioral Therapy; *CON*, Control; *CT*, Cognitive Therapy; *MC*, Mindfulness-basedCognitive Therapy; *NF*, Neurofeedback; *NIBS*, Noninvasive Brain Stimulation; *PE*, Psychoeducation; *SAT*, Self-Alert Training; *Taichi*; *VMT*, Vitamin–mineral treatment; *WMT*, Working Memory Training. *NA*, no available data.

**Table 2:** League table of the FU of ADHD core symptom.

FU of Core symptom	Comparison of treatments: Standardized Mean different(95%confidence intervals)/Effect of intervention in each row compared with intervention in each column					
**CBT**	NA	-1.75 (-5.08, 1.58)	NA	NA	NA	**-3.69 (-4.77,-2.61)**
0.92 (-1.71, 3.55)	**MC**	NA	NA	-0.42 (-3.75, 2.90)	NA	**-4.53 (-6.94,-2.12)**
**-2.50 (-4.61,-0.39)**	**-3.42 (-6.57,-0.27)**	**NF**	NA	NA	NA	-0.69 (-3.19, 1.81)
0.39 (-2.06, 2.85)	-0.53 (-3.81, 2.74)	2.89 (-0.12, 5.90)	**NIBS**	NA	NA	**-4.00 (-6.22,-1.78)**
0.50 (-3.74, 4.74)	-0.42 (-3.75, 2.90)	3.00 (-1.58, 7.58)	0.11 (-4.56, 4.78)	**PE**	NA	NA
-1.66 (-5.22, 1.91)	-2.58 (-6.75, 1.59)	0.84 (-3.13, 4.81)	-2.05 (-6.11, 2.02)	-2.16 (-7.49, 3.18)	**SAT**	-1.95 (-5.36, 1.46)
**-3.61 (-4.66,-2.56)**	**-4.53 (-6.94,-2.12)**	-1.11 (-3.14, 0.92)	**-4.00 (-6.22,-1.78)**	**-4.11 (-8.21, -0.00)**	-1.95 (-5.36, 1.46)	**CON**

Data are expressed as standardized mean differences (95%CI) between therapies. Significant results are highlighted in bold. Negative values favor the therapy in the row, and positive values favor the therapy in the column. Non-pharmacological therapies are listed in alphabetical order. The top section displays direct comparison results, while the bottom section shows results from mixed comparisons. *FU*, follow-up. *CBT*, Cognitive Behavioral Therapy; *CON*, Control; *MC*, Mindfulness-basedCognitive Therapy; *NF*, Neurofeedback; *NIBS*, Noninvasive Brain Stimulation; *PE*, Psychoeducation; *SAT*, Self-Alert Training; *VMT*, Vitamin–mineral treatment; *WMT*, Working Memory Training. *NA*, no available data.

### Secondary outcomes

#### Depression

For short-term depression efficacy (mostly self-assessed), CBT (SMD: -4.16, 95%CI: -5.51 to -2.81) and PE (SMD: -8.42, 95%CI: -12.26 to -4.59) were significantly superior to the control group, while MC (SMD: -0.80, 95%CI: -3.82 to 2.22), NF (SMD: 0.08, 95%CI: -3.34 to 3.50), NIBS (SMD: 0.07, 95%CI: -3.27 to 3.42), and SAT (SMD: 0.62, 95%CI: -4.12 to 5.36) showed no superiority. PE was significantly more effective than all other therapies in indirect comparisons. CBT outperformed MC (SMD: -3.36, 95%CI: -6.56 to -0.16), NF (SMD: -4.24, 95%CI: -7.66 to -0.81), and NIBS (SMD: -4.23, 95%CI: -7.84 to -0.62). For long-term effects, only CBT (SMD: -3.89, 95%CI: -5.95 to -1.83) remained superior to the control, with CBT also more effective than NF (SMD: -7.59, 95%CI: -11.77 to -3.40). Network plots and results are in **Supplementary Figures 1**-**4** and **Supplementary Tables 9**, **10**.

#### Anxiety

For short-term efficacy of anxiety, only CBT (SMD: -2.12, 95%CI: -3.18 to -1.07) was significantly superior to the control group. No significant differences were observed in the comparison of available non-pharmacological therapies. Regarding the long-term effect, the effects of CBT on anxiety were sustained over time. Furthermore, CBT (SMD: -7.25, 95%CI: -10.57 to -3.94) and MC (SMD: -6.10, 95%CI: -10.86 to -1.34) were significantly more effective than NF. NF demonstrated a significant negative effect (SMD: 3.87, 95%CI: 0.28 to 7.46). The network plots for anxiety and the results of the network meta-analyses are provided in **Supplementary Figures 5**-**8**, **Supplementary Tables 11** and **12**.

**Figure 4:**
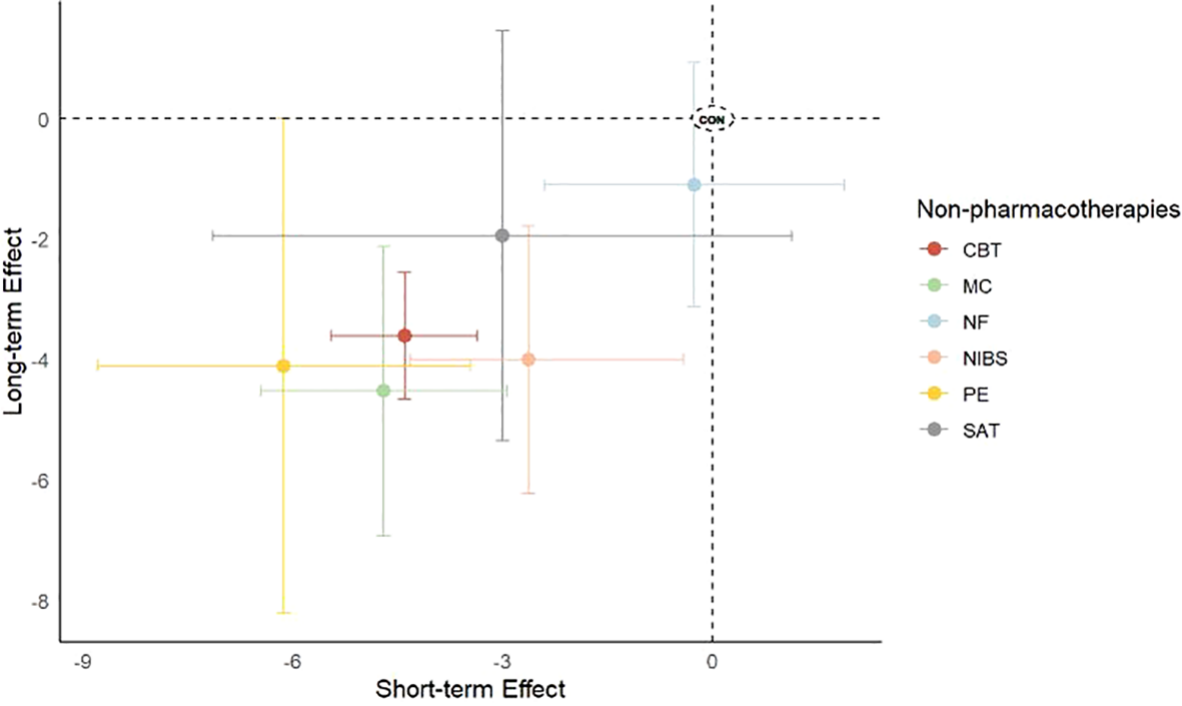
Two-dimensional graph of short-term versus long-term effect for core symptom. Effect sizes for individual therapies are represented by colored nodes, with bars indicating corresponding 95%CIs. The x-axis represents short-term effects, while the y-axis represents long-term effects. *CBT*, Cognitive Behavioral Therapy; *CON*, Control; *CT*, Cognitive Therapy; *MC*, Mindfulness-based cognitive Therapy; *NF*, Neurofeedback; *NIBS*, Noninvasive Brain Stimulation; *PE*, Psychoeducation; *SAT*, Self-Alert Training; *Taichi*; *VMT*, Vitamin–mineral treatment; *WMT*, Working Memory Training.

We presented these outcomes in a two-dimensional graph to better visualize the characteristics of a specific intervention’s effects (whether it is effective in the short term, long term, or both). For example, in **Figure 4**, we can observe that CBT, represented by red, shows significant effects in both short-term and long-term outcomes. Additional dimensional graphs are provided in **Supplementary Figures 9** and **10**.

### Heterogeneity and inconsistency

Heterogeneity was assessed using τ², I², and Q statistics, consistently indicating high variability among the studies (see **Supplementary Table 13**). Inconsistency analyses revealed no evidence of inconsistency among the studies included in the network meta-analysis (see **Supplementary Table 14**).

### Regression and subgroup analysis

In the regression analyses, we evaluated the impact of several variables, including publication year, geographical region, mean age, sample size, diagnostic criteria, scale type (rated by others versus self-reported), overall scale scores, and both the duration and frequency of the intervention (see **Supplementary Table 15**). Most findings from these analyses were robust; however, several covariates had a significant effect on outcomes. Consequently, subgroup analyses were performed, and the meta-analysis was re-executed focusing on these factors (see **Supplementary Table 16**).

CBT significantly improved depression when published in 2017 or later (SMD: -5.62, 95%CI: -7.46 to -3.79), but not before 2017 (SMD: -1.15, 95%CI: -2.45 to 0.16). It also reduced anxiety when the male proportion is 54% or higher (SMD: -5.23, 95%CI: -7.38 to -3.08), but not below 54% (SMD: -0.34, 95%CI: -1.66 to 0.99). NIBS improved ADHD core symptoms significantly when the total score is greater than or equal to 66 (SMD: -12.71, 95%CI: -23.10 to -2.33), but not when it is less than 66 (SMD: -1.29, 95%CI: -2.74 to 0.17). CBT showed a significant long-term effect on depression with a frequency equal to or greater than twice a week (SMD: -2.18, 95%CI: -2.68 to -1.67), but may worsen depression with fewer sessions (SMD: 2.62, 95%CI: 1.65 to 3.59).

### Risk of bias and certainty of evidence

The risk of bias was classified as low in 24.3% of the studies, unclear in 27%, and high in 48.6% (see **Supplementary Table 17**). According to the CINeMA assessment, the confidence in the evidence was “very low” or “low” for most remaining treatments (see **Supplementary Table 18**). Despite significant limitations due to the risk of bias and imprecision, the directness of the evidence and the low likelihood of publication bias provided assurance regarding the reliability of the findings. Funnel plots are shown in the **Supplementary Figure 11**.

### Publication bias

Regarding publication bias, Egger’s test revealed significant publication bias in the follow-up outcomes for ADHD core symptoms. We added three virtual studies using Duval’s trim and fill method and reran the meta-analysis for all studies. The results remained significant and did not reverse, indicating robust findings. The publication bias for all outcome measures is shown in **Supplementary Figures 12**-**17**.

## Discussion

This study presented a hierarchy of evidence for the short- and long-term effects of non-pharmacological treatments for adults with ADHD, with particular attention to the common comorbidities of anxiety and depression.

Overall, CBT was effective across all outcomes (both short-term and long-term) compared with the control group and is the most efficacious in reducing anxiety. PE, MC, CT and NIBS alleviated ADHD symptoms immediately post-intervention, with the effects of PE, MC, and NIBS being maintainable over time. Additionally, PE showed the most immediate improvement in depression symptoms post-intervention, although no long-term effects were observed. However, NF, SAT, Taichi, WMT, and VMT did not show any significant improvements, possibly due to the limited number of studies available.

Our results suggested that CBT is highly effective in improving the core symptoms of adults with ADHD, consistent with findings from previous reviews (28, 38). CBT demonstrated substantial efficacy in alleviating emotional symptoms both post-intervention and at follow-up, which aligns with prior research (39). Adults with ADHD often exhibit maladaptive cognitive patterns (40, 41) and harmful behavioral coping strategies (38), leading to feelings of frustration. These negative experiences may contribute to the development of anxiety and depression (42, 43). The primary goal of CBT is to assist individuals with ADHD in recognizing and modifying dysfunctional cognitions and maladaptive behaviors. It involves teaching cognitive and behavioral techniques that help individuals identify and manage challenges associated with deficits in attention, executive function, and inhibitory control (42, 44). By establishing new cognitive frameworks and problem-solving strategies, CBT mitigates the impact of ADHD symptoms on daily life, enhancing individuals’ perceptions of frustration and reducing levels of depression and anxiety. Furthermore, once adults with ADHD acquire these coping strategies, they may experience long-term benefits. Repeated application of these skills enables individuals to integrate CBT techniques into their personal circumstances, leading to sustained improvements in managing both core symptoms and emotional disorders. However, only six interventions were assessed for short-term anxiety impacts and five for long-term effects. Including studies with additional interventions (e.g., PE) could alter the hierarchy of effectiveness.

In our study, MC refers to mindfulness and mindfulness-related interventions. Dysfunction of the default mode network (DMN) is observed in adults with ADHD (45), which may affect attention and impulse control and is associated with mind-wandering. Spontaneous mind-wandering is a central feature of ADHD symptomatology (46) and may underlie many functional impairments of ADHD (46, 47). In contrast to CBT, MC focuses on participants’ present-moment experiences and thoughts (48), helping individuals learn to perceive and observe their thoughts and feelings nonjudgmentally (49). Research indicates that MC help adults with ADHD reduce attention distraction and mind-wandering by improving the functioning of the DMN, thereby enhancing attention and other cognitive functions, such as impulse control and working memory (20). This improvement in DMN functioning may explain why MC was effective in improving core symptoms of ADHD, consistent with findings from other reviews (50, 51). Furthermore, MC demonstrated the best performance in maintaining long-term effects. However, our results did not reveal significant effects on depression and anxiety, which stands in contrast to the findings reported in previous meta-analyses by Kretschmer et al. (52). Differences in the study inclusion and the data analysis methods may account for the divergent results.

Apart from its long-term effects on depression, PE significantly improved ADHD core symptoms. However, no data was available to assess its effects on anxiety. Additionally, PE demonstrated the best short-term improvements in ADHD core symptoms and depression. Unlike other psychological interventions, PE focuses on educating patients about their disorder (e.g., causes, symptoms, and treatment options) while enhancing compliance, self-esteem, and mutual support in managing everyday challenges (53). This approach may be more effective for adults with ADHD, as they can better understand and accept their condition, reducing the subjective impact on their lives. PE may also resonate more with practical life experiences, facilitating its application. However, there is a lack of RCTs on PE for adults with ADHD, and no relevant meta-analyses exist. A recent scoping review (54) summarized the PE’s definitions and themes but did not assess its effectiveness. The authors highlighted a significant issue: the problem of demarcation. PE is often considered part of ADHD counselling or other interventions such as CBT, making it difficult to determine whether a given intervention qualifies as PE. Additionally, the term “PE” is sometimes used without clear explanations, leading to misunderstanding and confusion. The limited studies and inconsistent definitions make it difficult to draw definitive conclusions. While our network meta-analysis results are based on direct comparisons with MC and CBT (19, 55, 56), they are drawn from only three studies and should be interpreted with caution.

NIBS refers to techniques like transcranial magnetic stimulation(TMS) and transcranial direct current stimulation (tDCS) that modulate brain excitability (57). NIBS can modulate neural activity and induce neuroplasticity, which underlies its efficacy in improving the core symptoms of ADHD. Similar findings have been reported in the literature (58). However, research on the effects of NIBS on ADHD has shown inconsistent results. One meta-analysis (59) involving both children and adults found that tDCS applied to the left or bilateral dorsolateral prefrontal cortex (dlPFC) improved inhibition and processing speed, but not attention. Another meta-analysis (60) focused on children found that tDCS improved overall symptom severity, inattention, and impulsivity, but not hyperactivity. This discrepancy may be related to individual neuropsychological and anatomical differences across populations, highlighting the need for more RCTs.

Notably, statistical results indicated that neurofeedback (NF) showed a significant negative impact on anxiety levels at follow-up, suggesting that this intervention may exacerbate anxiety symptoms in adults with ADHD during the follow-up period. However, NF was not directly compared with the control group, the NMA results were based on a comparison between NF and CBT. In this direct comparison, CBT was observed to be significantly more effective than NF, which could influence the interpretation of NF’s impact relative to the control group and affect the conclusions drawn.

The regression and subgroup analyses suggested that the publication year, proportion of males, total score of the core symptoms, intervention frequency and follow-up lengths may influence the results. The variability in follow-up lengths is an important factor to consider when interpreting our findings on the long-term effects of non-pharmacological therapies. Despite efforts to select comparable follow-up periods during data extraction, substantial variation remains, ranging from 3 to 48 weeks (see **Supplementary Table 8**). Follow-up length may be influenced by intervention characteristics, study design, and objectives, so it was included as a covariate in the regression analysis. The results show that follow-up length significantly impacts meta-analysis outcomes. A subgroup analysis comparing follow-up durations of less than 12 weeks versus 12 weeks or more revealed limited analysis depth due to differing intervention methods between subgroups, with only one group having a follow-up of less than 12 weeks. These differences highlight the variability in follow-up periods across interventions, a factor that is also observed in real-world settings. Notably, the limited number of studies included in the subgroup analysis, the scarcity of direct comparisons, and the lack of consistency in intervention methods across groups must also be taken into account.

In summary, considering both short-term and long-term effects, evidence from this network meta-analysis supports MC as the first-choice non-pharmacological therapy for adults with ADHD without comorbid anxiety or depression and CBT as the first-line non-pharmacological treatment for adults with ADHD comorbid with anxiety or depression, addressing both core symptoms and emotional disorders. Although PE demonstrates better short-term efficacy in improving core symptoms of ADHD and alleviating depression compared to MC and CBT, its long-term effects appear limited, and the evidence base remains insufficient. While we do not advocate for PE as a primary intervention, its potential merits justify further rigorous research to explore its effectiveness and optimize its application.

## Limitations

Our study has several limitations. First, the included studies primarily compared non-pharmacological treatments with control groups or conditions (e.g., waiting lists or treatment as usual), with limited direct comparisons between different non-pharmacological treatments.

Second, we observed significant statistical heterogeneity in the network meta-analysis. Despite conducting rigorous regression and subgroup analyses to explore heterogeneity, results indicate that some factors may influence the outcomes. Further analysis was constrained due to the limited number of studies for each intervention.

Third, certain interventions in our network are represented by only one or two studies (e.g., one study for Tai Chi and two studies for WMT). In such cases, the impact of expectancy effects and the variability in study quality can substantially influence the evaluation of an intervention’s efficacy. Although studies attempted to mitigate these effects by employing active and semi-active control groups, this remains a notable limitation.

## Conclusion

This meta-analysis confirms that non-pharmacological treatments offer a potential alternative for managing ADHD with CBT emerging as the most preferable option for core symptoms and co-occurring depression and anxiety, both post-intervention and at follow-up. MC and PE demonstrated favorable effects on core ADHD symptoms, but PE was only included in three studies. The observed effects may be influenced by publication year, male participant proportion, and intervention frequency and duration. These findings provide practical insights for designing non-pharmacological or multidisciplinary interventions to manage ADHD core symptoms and co-occurring emotional disorders. Overall, the existing literature on non-pharmacological treatments mainly focuses on CBT, and many studies are of low quality, and lacking robust evidence. Caution is needed in interpreting NMA results, and high-quality RCTs are urgently required for more reliable insights.

## Data Availability

The original contributions presented in the study are included in the article/**Supplementary Material**. Further inquiries can be directed to the corresponding authors.
