# Supplementary material for: Short-term and long-term effect of non-pharmacotherapy for adults with ADHD: a systematic review and network meta-analysis
Source: Front Psychiatry. 2025 Jan 31;16:1516878. doi: 10.3389/fpsyt.2025.1516878 (PMC11825462; doi:10.3389/fpsyt.2025.1516878)
Supplement: Supplementary file 1 [file DataSheet1.pdf]

Supplementary Figure

CONTENT 1

Figure 1: Network plot of depression 2

Figure 2: Network plot of follow-up of depression 3

Figure 3: Forest plot of depression 4

Figure 4: Forest plot of follow-up of depression 5

Figure 5: Network plot of anxiety 6

Figure 6: Network plot of follow-up of anxiety 7

Figure 7: Forest plot of anxiety 8

Figure 8: Forest plot of follow-up of anxiety 9

Figure 9: Two-dimensional graph of depression 10

Figure 10: Two-dimensional graph of anxiety 11

Figure 11: Summary of limitations of included studies (CINeMA) 12

Figure 12: Publication bias of ADHD core symptom 13

Figure 13: Publication bias of depression 14

Figure 14: Publication bias of anxiety 15

Figure 15: Publication bias of the follow-up of ADHD core symptom 16

Figure 16: Publication bias of the follow-up of depression 17

Figure 17: Publication bias of the follow-up of anxiety 18

Figure 1: Network plot of depression

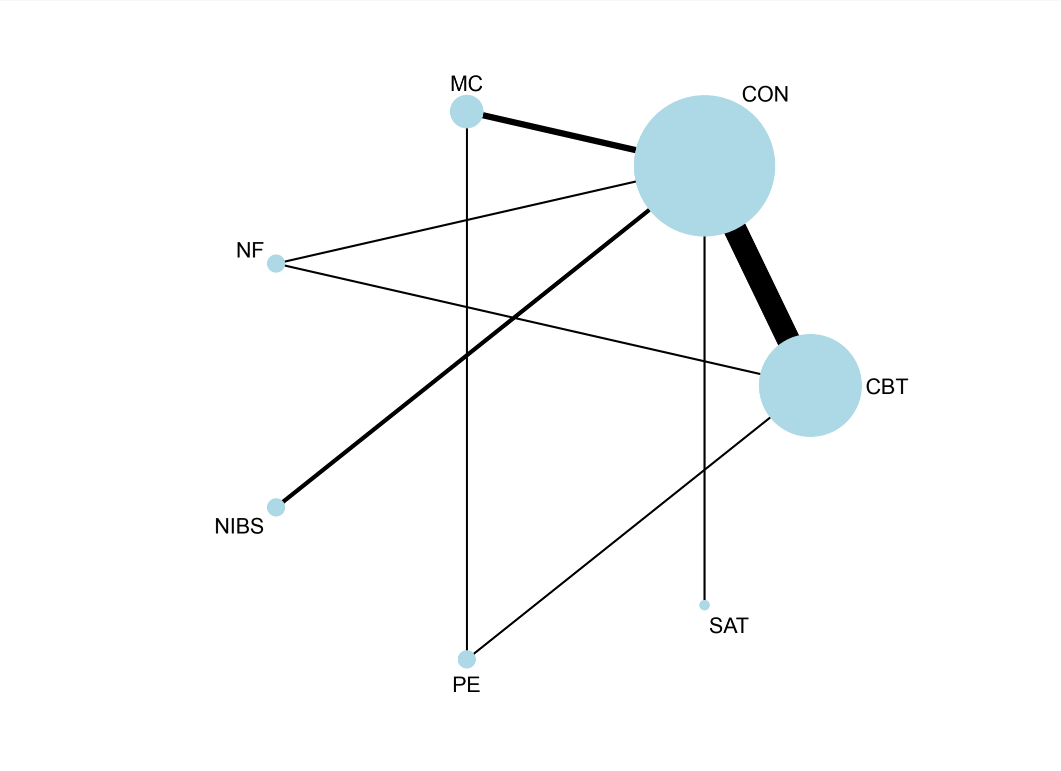

Figure 1. Network plot of depression. The width of the lines is proportional to the number of trials comparing each pair of treatments, and the size of each circle is proportional to the sample size. *CON*: Control Intervention; *CBT*: Cognitive Behavioral Therapy; *MC*: Mindfulness-based Cognitive Therapy; *NF*: Neurofeedback; *NIBS*: Noninvasive Brain Stimulation; *PE*: Psychoeducation; *SAT:* Self-Alert Training.

Figure 2: Network plot of follow-up of depression

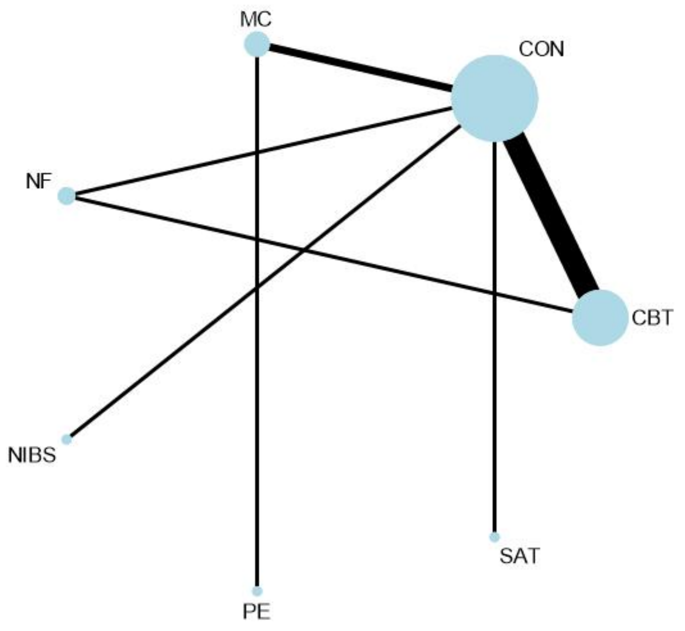

Figure 2. Network plot of the follow-up of depression. The width of the lines is proportional to the number of trials comparing each pair of treatments, and the size of each circle is proportional to the sample size. *CON*: Control Intervention; *CBT*: Cognitive Behavioral Therapy; *MC*: Mindfulness-based Cognitive Therapy; *NF*: Neurofeedback; *NIBS*: Noninvasive Brain Stimulation; *PE*: Psychoeducation; *SAT*: Self-Alert Training.

Figure 3: Forest plot of depression

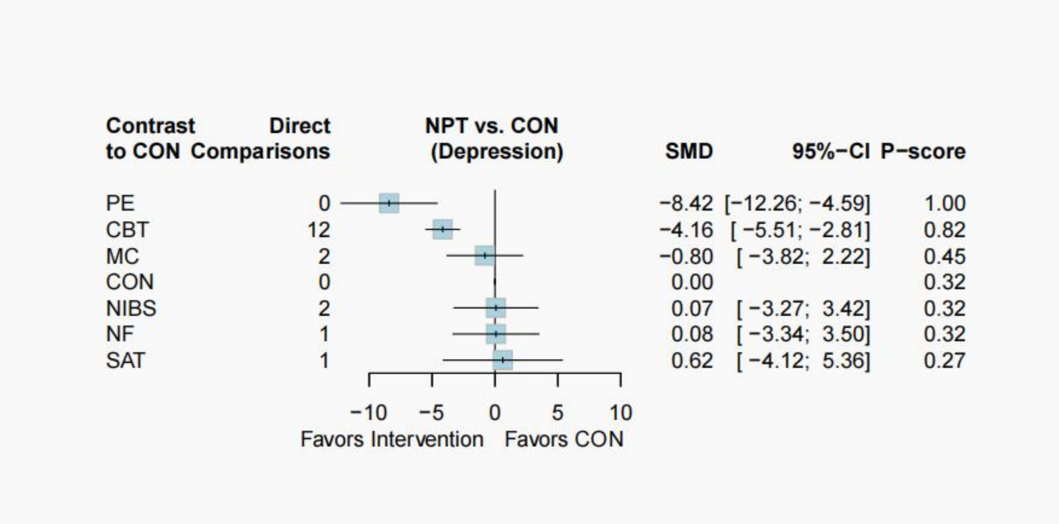

Figure 3: The forest plot includes all eligible trials comparing efficacy against the control group (CON). *NPT*: Non-pharmacological therapy; *CBT*: Cognitive Behavioral Therapy; *MC*: Mindfulness-based Cognitive Therapy; *NF*: Neurofeedback; *NIBS*: Noninvasive Brain Stimulation; *PE*: Psychoeducation; *SAT*: Self-Alert Training. *SMD:* standardized mean difference.

Figure 4: Forest plot of follow-up of depression

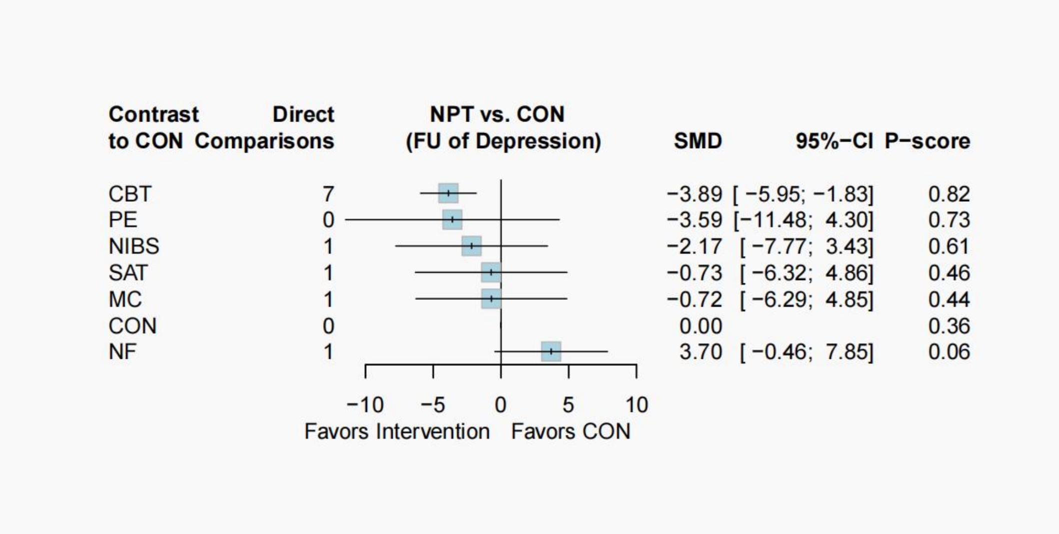

Figure 4. The forest plot includes all eligible trials comparing efficacy against the control group (CON). *NPT*: Non-pharmacological therapy. *CBT*: Cognitive Behavioral Therapy; *MC*: Mindfulness-based Cognitive Therapy; *NF*: Neurofeedback; *NIBS*: Noninvasive Brain Stimulation; *PE*: Psychoeducation; *SAT*: Self-Alert Training. *SMD:* standardized mean difference.

Figure 5: Network plot of anxiety

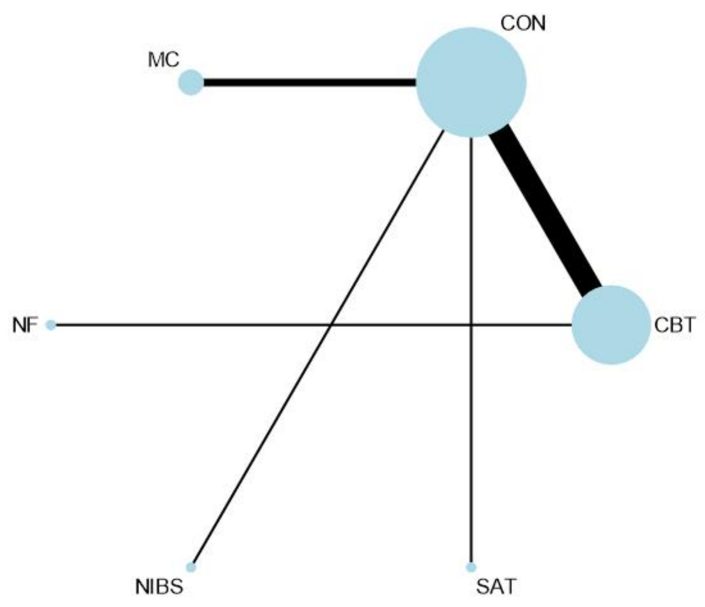

Figure 5. Network plot of anxiety. The width of the lines is proportional to the number of trials comparing each pair of treatments, and the size of each circle is proportional to the sample size. *CON*: Control Intervention; *CBT*: Cognitive Behavioral Therapy; *MC*: Mindfulness-based Cognitive Therapy; *NF*: Neurofeedback; *NIBS*: Noninvasive Brain Stimulation; *SAT:* Self-Alert Training.

Figure 6: Network plot of follow-up of anxiety

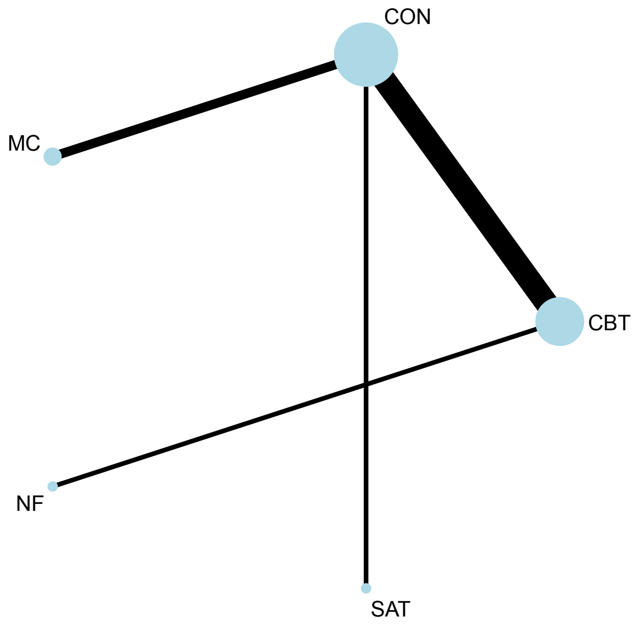

Figure 6. Network plot of the follow-up of anxiety. The width of the lines is proportional to the number of trials comparing each pair of treatments, and the size of each circle is proportional to the sample size. *CON*: Control Intervention; *CBT*: Cognitive Behavioral Therapy; *MC*: Mindfulness-based Cognitive Therapy; *NF*: Neurofeedback; *SAT:* Self-Alert Trainin

Figure 7: Forest plot of anxiety

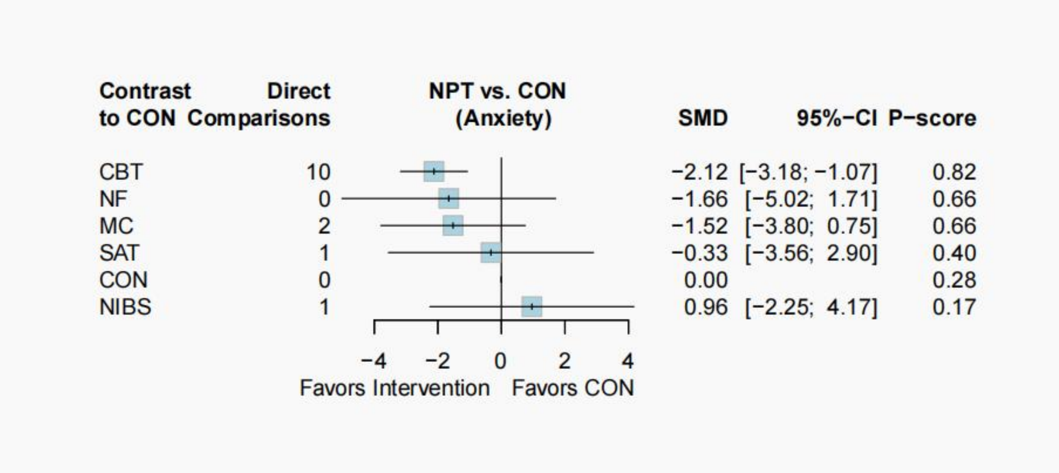

Figure 7. The forest plot includes all eligible trials comparing efficacy against the control group (CON). *NPT*: Non-pharmacological therapy. *CBT*: Cognitive Behavioral Therapy; *MC*: Mindfulness-based Cognitive Therapy; *NF*: Neurofeedback; *NIBS*: Noninvasive Brain Stimulation; *SAT*: Self-Alert Training. *SMD:* standardized mean difference.

Figure 8: Forest plot of follow-up of anxiety

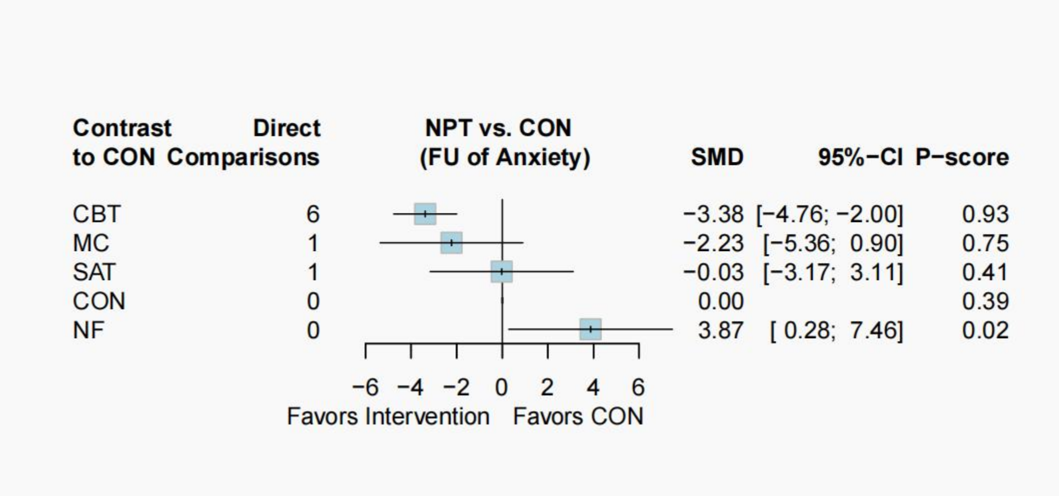

Figure 8. The forest plot includes all eligible trials comparing efficacy against the control group (CON). *NPT*: Non-pharmacological therapy. *CBT*: Cognitive Behavioral Therapy; *MC*: Mindfulness-based Cognitive Therapy; *NF*: Neurofeedback; *SAT*: Self-Alert Training. *SMD:* standardized mean difference.

Figure 9: Two-dimensional graph of depression

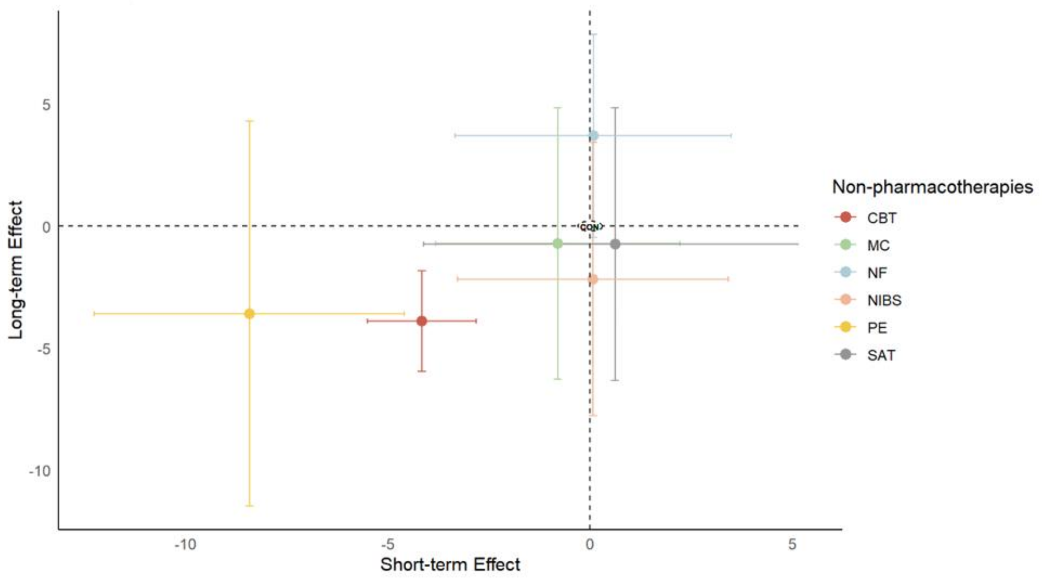

Figure 9. Two-dimensional graph of short-term versus long-term effect for depression.

Effect sizes for individual therapies are represented by coloured nodes, with bars indicating corresponding 95% CIs. The x-axis represents short-term effects, while the y-axis represents long-term effects. *CBT*: Cognitive Behavioral Therapy; *MC*: Mindfulness-based Cognitive Therapy; *NF*: Neurofeedback; *NIBS*: Noninvasive Brain Stimulation; *PE*: Psychoeducation; *SAT*: Self-Alert Training.

Figure 10: Two-dimensional graph of anxiety

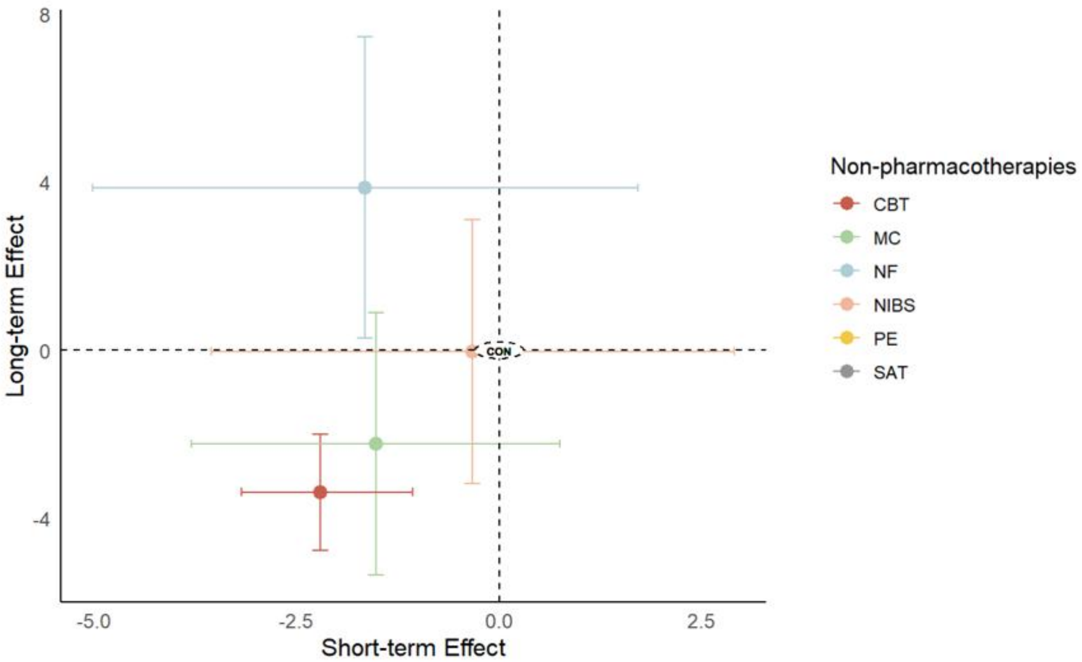

Figure 10. Two-dimensional graph of short-term versus long-term effect for anxiety.

Effect sizes for individual therapies are represented by coloured nodes, with bars indicating corresponding 95% CIs. The x-axis represents short-term effects, while the y-axis represents long-term effects. *CBT*: Cognitive Behavioral Therapy; *MC*: Mindfulness-based Cognitive Therapy; *NF*: Neurofeedback; *NIBS*: Noninvasive Brain Stimulation; *PE*: Psychoeducation; *SAT*: Self-Alert Training.

Figure 11: Summary of limitations of included studies (CINeMA)

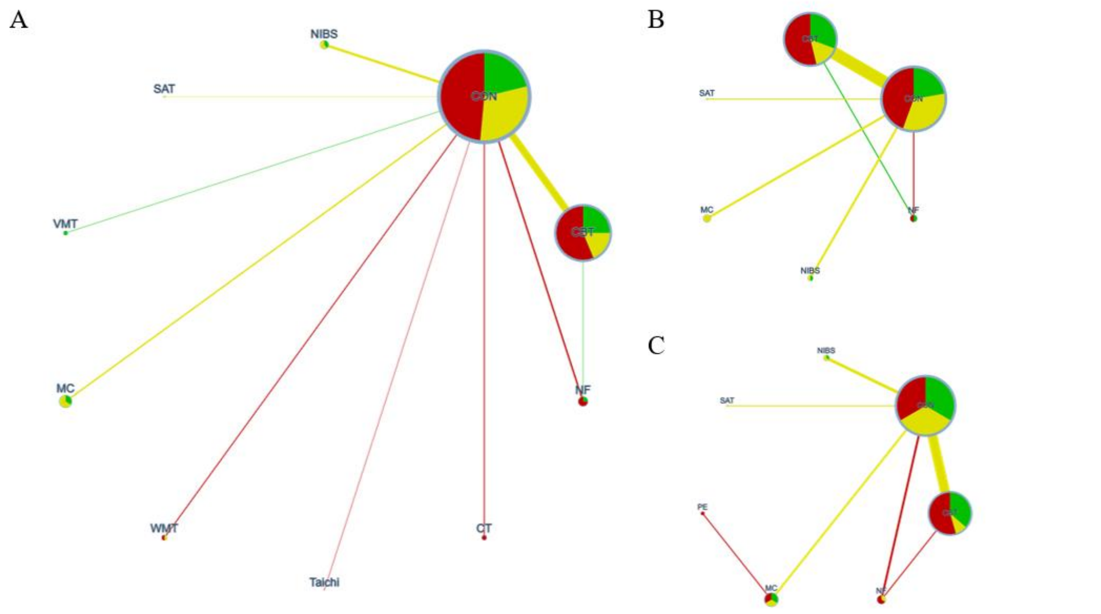

Figure 11. Summary of limitations of included studies. (A) Core Symptom; (B) Depression; (C) Follow-up of Core Symptom. Based on the recommendations of the CINeMA online document (https://cinema.ispm.unibe.ch/), we only graded the results of ADHD core symptoms, depression and follow-up of core symptoms whether each module needs to be downgraded according to the following criteria. We classified the quality evaluation results of each included study into low-risk, some concern and high-risk. We selected the rule is average RoB2. No need to downgrade when the result is "no concerns", downgrade one level when "some concerns" and downgrade two when "major concerns".

Figure 12: Publication bias of ADHD core symptom

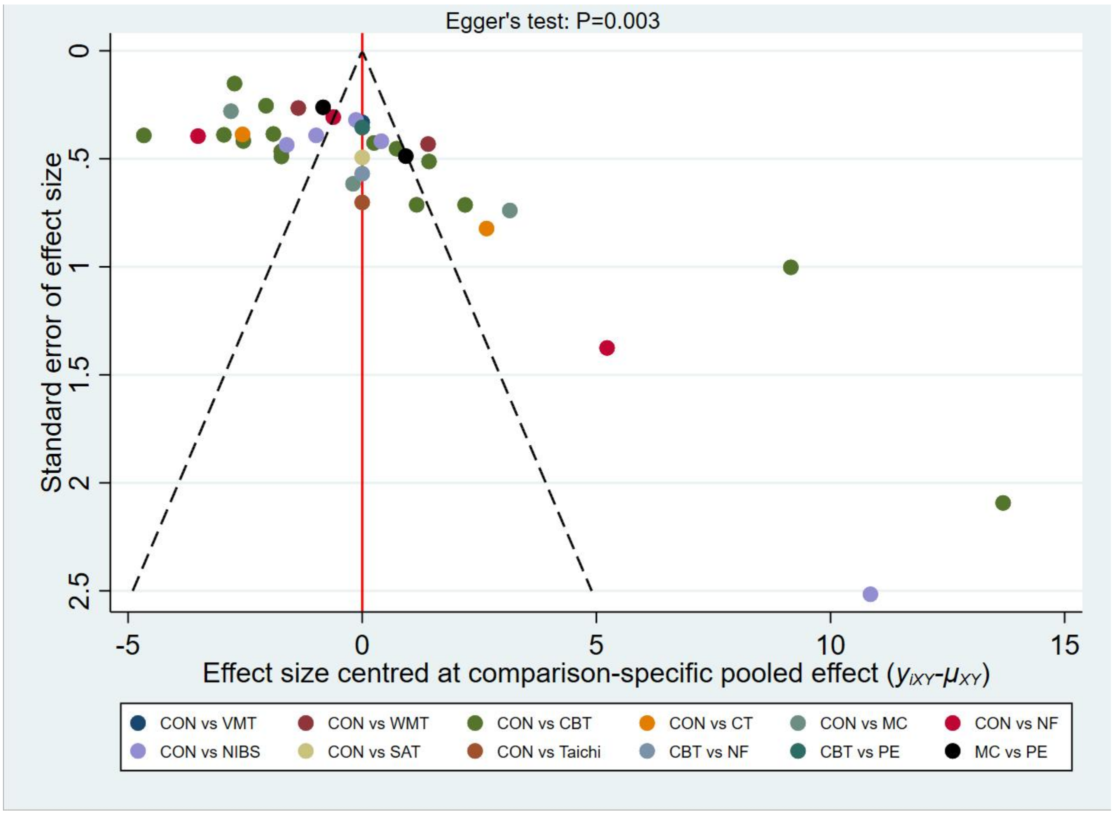

Figure 12. The funnel plot of change of core symptoms of all non-pharmacological interventions compared to the control group. *CON*: Control Intervention; *CBT*: Cognitive Behavioral Therapy; *CT*: Cognitive Therapy; *MC*: Mindfulness-based Cognitive Therapy; *NF*: Neurofeedback; *NIBS*: Noninvasive Brain Stimulation; *PE*: Psychoeducation; *SAT*: Self-Alert Training; *Taichi*; *VMT*: Vitamin–mineral treatment; *WMT*: Working Memory Training. As shown in the figure, the asymmetry of the funnel plot and the P value of Egger's test might indicate publication bias in the analysis. However, there were no indications of publication with the Duval's trim and fill method (no new studies added or excluded).

Figure 13: Publication bias of depression

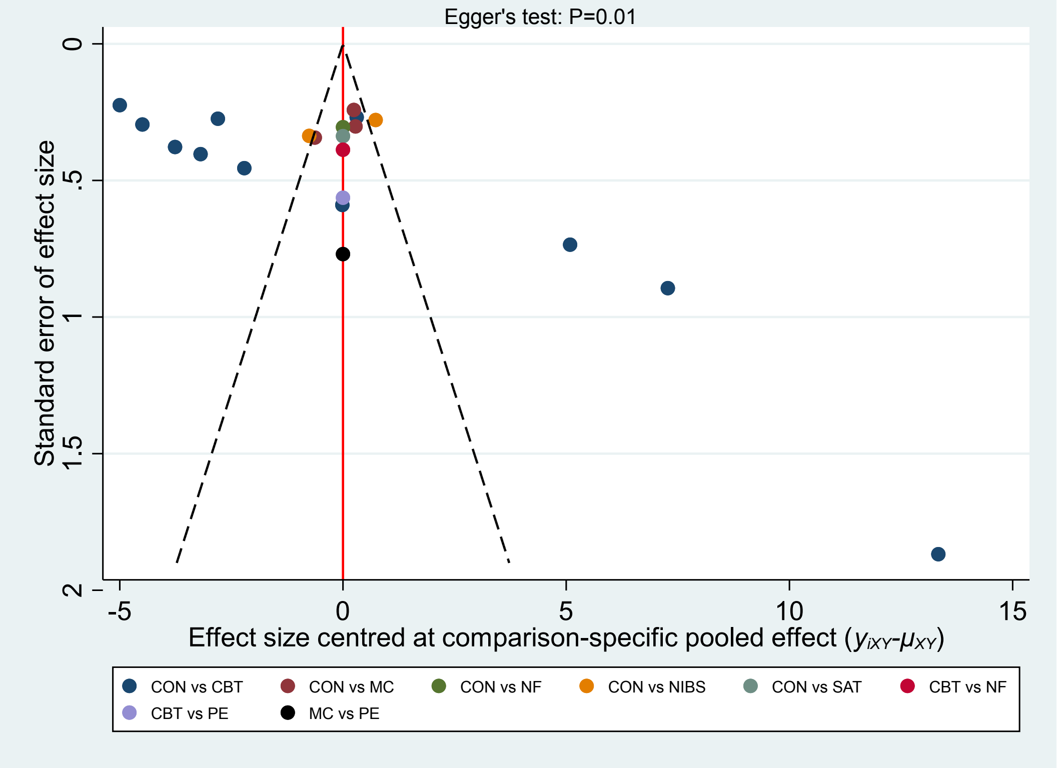

Figure 13. The funnel plot of change of depression of all non-pharmacological interventions compared to the control group. *CON*: Control Intervention; *CBT*: Cognitive Behavioral Therapy; *MC*: Mindfulness-based Cognitive Therapy; *NF*: Neurofeedback; *NIBS*: Noninvasive Brain Stimulation; *PE*: Psychoeducation; *SAT*: Self-Alert Training. As shown in the figure, the asymmetry of the funnel plot and the P value of Egger's test might indicate publication bias in the analysis. However, there were no indications of publication with the Duval's trim and fill method (no new studies added or excluded).

Figure 14: Publication bias of anxiety

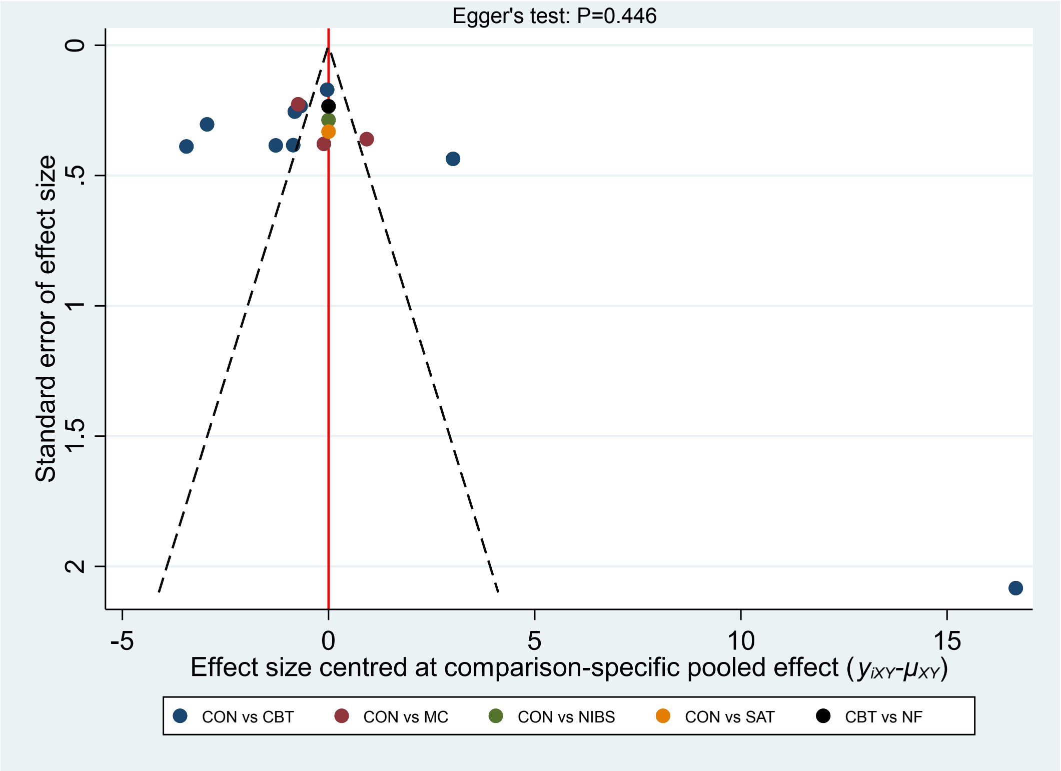

Figure 14. The funnel plot of change in the anxiety of all non-pharmacological interventions compared to the control group. *CON*: Control Intervention; *CBT*: Cognitive Behavioral Therapy; *MC*: Mindfulness-based Cognitive Therapy; *NF*: Neurofeedback; *NIBS*: Noninvasive Brain Stimulation; *SAT*: Self-Alert Training. As shown in the figure, the funnel plot had good symmetry. Therefore, no small study effect was found for the primary outcome.

Figure 15: Publication bias of the follow-up of ADHD core symptom

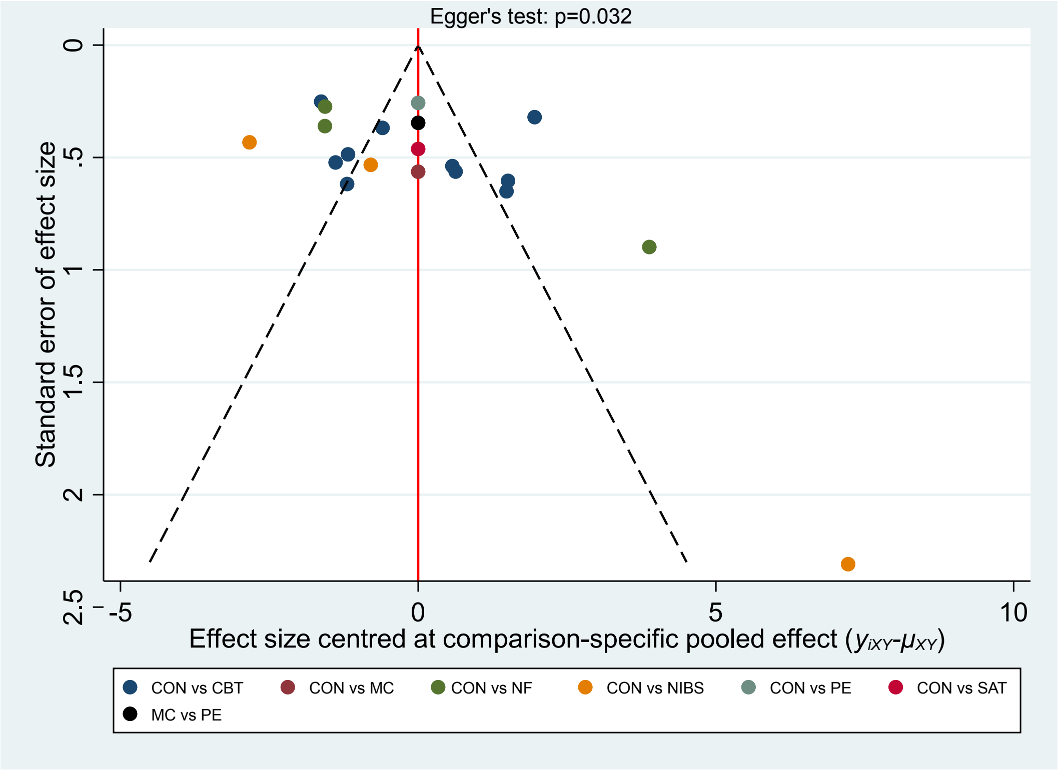

Figure 15. The funnel plot of change in the follow-up of ADHD core symptom of all non-pharmacological interventions compared to the control group. *CON*: Control Intervention; *CBT*: Cognitive Behavioral Therapy; *CT*: Cognitive Therapy; *MC*: Mindfulness-based Cognitive Therapy; *NF*: Neurofeedback; *NIBS*: Noninvasive Brain Stimulation; *PE*: Psychoeducation; *SAT*: Self-Alert Training. As shown in the figure, the asymmetry of the funnel plot and the P value of Egger's test might indicate publication bias in the analysis. Duval's trim and fill method filled 3 virtual studies and reran the Meta-analysis for all studies, the result remained significant and did not reverse, indicating robust results.

Figure 16: Publication bias of the follow-up of depression

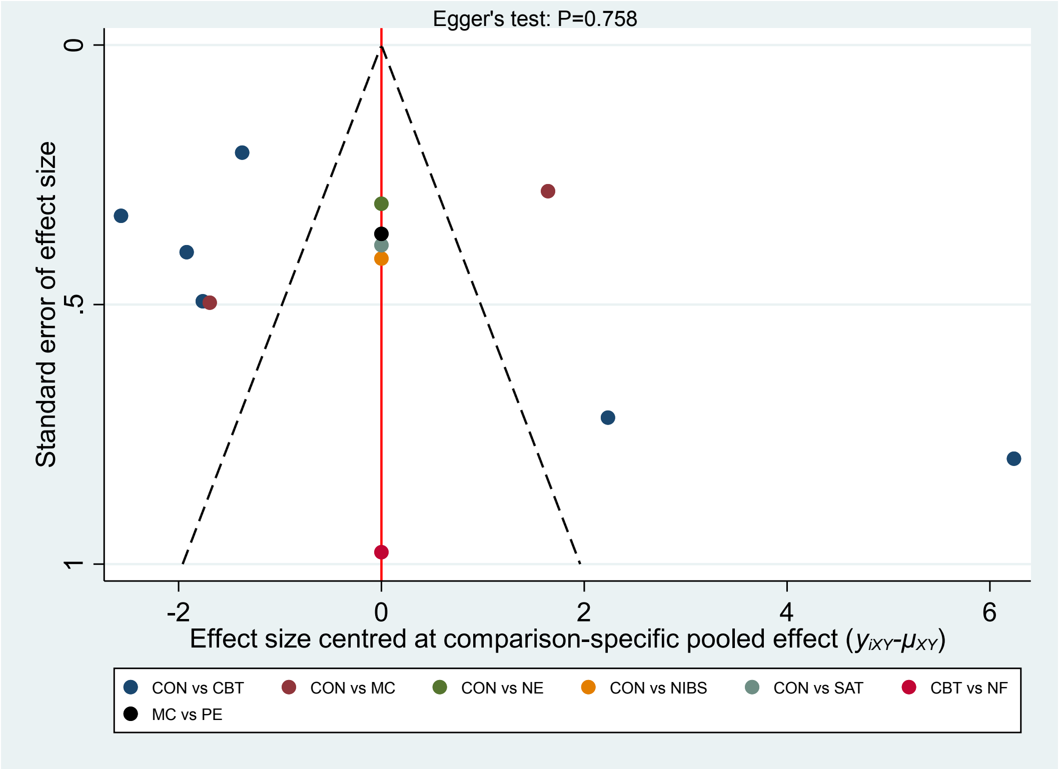

Figure 16: The funnel plot of change in the follow-up of depression of all non-pharmacological interventions compared to the control group. *CON*: Control Intervention; *CBT*: Cognitive Behavioral Therapy; *CT*: Cognitive Therapy; *MC*: Mindfulness-based Cognitive Therapy; *NF*: Neurofeedback; *NIBS*: Noninvasive Brain Stimulation; *PE*: Psychoeducation; *SAT*: Self-Alert Training. As shown in the figure, the funnel plot had good symmetry. Therefore, no small study effect was found for the primary outcome.

Figure 17: Publication bias of the follow-up of anxiety

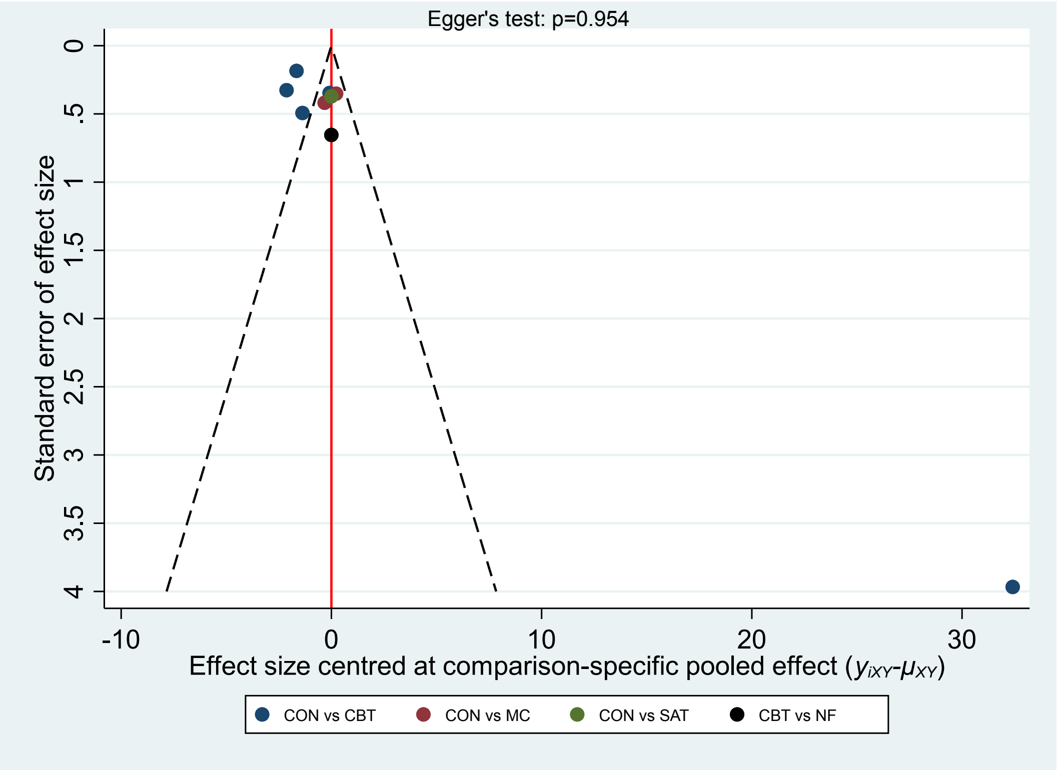

Figure 17. The funnel plot of change in the follow-up of anxiety of all non-pharmacological interventions compared to the control group. *CON*: Control Intervention; *CBT*: Cognitive Behavioral Therapy; *CT*: Cognitive Therapy; *MC*: Mindfulness-based Cognitive Therapy; *NF*: Neurofeedback; *NIBS*: Noninvasive Brain Stimulation; *PE*: Psychoeducation; *SAT*: Self-Alert Training. As shown in the figure, the funnel plot had good symmetry. Therefore, no small study effect was found for the primary outcome.
