# Supplementary material for: Short-term and long-term effect of non-pharmacotherapy for adults with ADHD: a systematic review and network meta-analysis
Source: Front Psychiatry. 2025 Jan 31;16:1516878. doi: 10.3389/fpsyt.2025.1516878 (PMC11825462; doi:10.3389/fpsyt.2025.1516878)
Supplement: Supplementary file 2 [file Table1.docx]

***Supplementary Table***

[**CONTENT** 1](#_Toc186155703)

[TABLE 1: Search Strategy for the PubMed Database 2](#_Toc186155704)

[TABLE 2: Search Strategy for the Web of Science Database 3](#_Toc186155705)

[TABLE 3: Search Strategy for the Cochrane Database 4](#_Toc186155706)

[TABLE 4: Search Strategy for the EMBASE Database 5](#_Toc186155707)

[TABLE 5: Inclusion and Exclusion Criteria 6](#_Toc186155708)

[TABLE 6: Rating Scales Incorporated 7](#_Toc186155709)

[TABLE 7: Definitions of Non-pharmacotherapies and Control Group/Condition 8](#_Toc186155710)

[TABLE 8: Characteristics of Studies Included 11](#_Toc186155711)

[TABLE 9: The League Table of Depression 15](#_Toc186155712)

[TABLE 10: The League Table of Follow-up of Depression 16](#_Toc186155787)

[TABLE 11: The League Table of Anxiety 17](#_Toc186155788)

[TABLE 12: The League Table of the Follow-up of Anxiety 18](#_Toc186155831)

[TABLE 13: Evaluation of Heterogeneity 19](#_Toc186155832)

[TABLE 14: Evaluation of Inconsistency 20](#_Toc186155833)

[TABLE 15: Outcome of Network Meta-Regression 21](#_Toc186155834)

[TABLE 16: Subgroup Analysis 22](#_Toc186155835)

[TABLE 17: The Risk of Bias Assessment for The Individual Included Studies 25](#_Toc186155836)

[TABLE 18: The Overall Certainty of Evidence (CINeMA) 27](#_Toc186155837)

## TABLE 1: Search Strategy for the PubMed Database

| **Step** | **Search strategy** |
| --- | --- |
| #1 | Search: "Attention Deficit Disorder with Hyperactivity" [Mesh] |
| #2 | Search: "Adult" [Mesh] |
| #3 | Search: "Randomized Controlled Trial" [Publication Type] |
| #4 | Search: "Controlled Clinical Trial" [Publication Type] |
| #5 | Search: Stimulants [Title/Abstract] |
| #6 | Search: Non-stimulants [Title/Abstract] |
| #7 | Search: methylphenidate [Title/Abstract] |
| #8 | Search: amphetamine [Title/Abstract] |
| #9 | Search: atomoxetine [Title/Abstract] |
| #10 | Search: guanfacine [Title/Abstract] |
| #11 | Search: clonidine [Title/Abstract] |
| #12 | Search: antidepressants [Title/Abstract] |
| #13 | Search: Antipsychotics [Title/Abstract] |
| #14 | Search: ("Attention Deficit Disorder with Hyperactivity" [Mesh]) AND ("Adult" [Mesh]) |
| #15 | Search: ("Randomized Controlled Trial" [Publication Type]) OR ("Controlled Clinical Trial" [Publication Type]) |
| #16 | Search: "Drug Therapy/methods"[Mesh] |
| #17 | ((((((((Stimulants [Title/Abstract]) OR (Non-stimulants [Title/Abstract])) OR (methylphenidate [Title/Abstract])) OR (amphetamine [Title/Abstract])) OR (atomoxetine [Title/Abstract])) OR (guanfacine [Title/Abstract])) OR (clonidine [Title/Abstract])) OR (antidepressants [Title/Abstract])) OR (Antipsychotics [Title/Abstract]) |
| #18 | ("Drug Therapy/methods"[Mesh]) OR (((((((((Stimulants [Title/Abstract]) OR (Non-stimulants [Title/Abstract])) OR (methylphenidate [Title/Abstract])) OR (amphetamine [Title/Abstract])) OR (atomoxetine [Title/Abstract])) OR (guanfacine [Title/Abstract])) OR (clonidine [Title/Abstract])) OR (antidepressants [Title/Abstract])) OR (Antipsychotics [Title/Abstract])) |
| #19 | (("Attention Deficit Disorder with Hyperactivity" [Mesh]) AND ("Adult" [Mesh])) AND (("Randomized Controlled Trial" [Publication Type]) OR ("Controlled Clinical Trial" [Publication Type])) |
| #20 | ((("Attention Deficit Disorder with Hyperactivity" [Mesh]) AND ("Adult" [Mesh])) AND (("Randomized Controlled Trial" [Publication Type]) OR ("Controlled Clinical Trial" [Publication Type]))) NOT (((((((((Stimulants [Title/Abstract]) OR (Non-stimulants [Title/Abstract])) OR (methylphenidate [Title/Abstract])) OR (amphetamine [Title/Abstract])) OR (atomoxetine [Title/Abstract])) OR (guanfacine [Title/Abstract])) OR (clonidine [Title/Abstract])) OR (antidepressants [Title/Abstract])) OR (Antipsychotics [Title/Abstract])) |

## TABLE 2: Search Strategy for the Web of Science Database

| **Step** | **Search strategy** |
| --- | --- |
| #1 | TS=("Attention Deficit Disorders with Hyperactivity" OR ADHD OR "Attention Deficit Hyperactivity Disorder" OR "Hyperkinetic Syndrome" OR "Syndromes, Hyperkinetic" OR "Attention Deficit-Hyperactivity Disorder" OR "Attention Deficit-Hyperactivity Disorders" OR "Deficit-Hyperactivity Disorder, Attention" OR "Deficit-Hyperactivity Disorders, Attention" OR "Disorder, Attention Deficit-Hyperactivity" OR "Disorders, Attention Deficit-Hyperactivity" OR ADDH OR "Attention Deficit Hyperactivity Disorders" OR "Attention Deficit Disorder" OR "Attention Deficit Disorders" OR "Deficit Disorder, Attention" OR "Deficit Disorders, Attention" OR "Disorder, Attention Deficit" OR "Disorders, Attention Deficit") |
| #2 | TS=(RCT OR "randomized controlled trial" OR "randomised controlled trial" OR "controlled clinical trial" OR "Clinical Trials, Randomized" OR "Trials, Randomized Clinical" OR "Controlled Clinical Trials, Randomized") |
| #3 | TS=(adult) |
| #4 | TS=("drug therapy" OR "pharmacologic therapy" OR "pharmacotherapy" OR "chemotherapy") |
| #5 | TS=(Stimulants OR Non-stimulants OR methylphenidate OR amphetamine OR atomoxetine OR guanfacine OR clonidine OR antidepressants OR Antipsychotic) |
| #6 | #4 OR #5 |
| #7 | #1 AND #2 AND #3 |
| #8 | #7 NOT #6 |

## TABLE 3: Search Strategy for the Cochrane Database

| **Step** | **Search strategy** |
| --- | --- |
| #1 | MeSH descriptor: [Attention Deficit Disorder with Hyperactivity] explode all trees |
| #2 | MeSH descriptor: [Adult] explode all trees |
| #3 | MeSH descriptor: [drug therapy] explode all trees |
| #4 | (Stimulants OR Non-stimulants OR methylphenidate OR amphetamine OR atomoxetine OR guanfacine OR clonidine OR antidepressants OR Antipsychotic): ti,ab,kw |
| #5 | #1 AND #2 |
| #6 | #3 OR #4 |
| #7 | #5 NOT #6 |

## TABLE 4: Search Strategy for the EMBASE Database

| **Step** | **Search strategy** |
| --- | --- |
| #1 | 'attention deficit hyperactivity disorder'/exp |
| #2 | 'adult'/exp |
| #3 | 'randomized controlled trial'/exp |
| #4 | 'drug therapy'/exp |
| #5 | (stimulants):ti,ab,kw OR (('non stimulants'):ti,ab,kw) OR ((methylphenidate):ti,ab,kw) OR ((amphetamine):ti,ab,kw) OR ((guanfacin):ti,ab,kw) OR ((clonidine):ti,ab,kw) OR ((antidepressants):ti,ab,kw) OR ((antipsychotics):ti,ab,kw) |
| #6 | #1 AND #2 AND #3 |
| #7 | #4 OR #5 |
| #8 | #6 NOT #7 |

## TABLE 5: Inclusion and Exclusion Criteria

| **Term** | **Inclusion** | **Exclusion** |
| --- | --- | --- |
| Population | -Adults older than 18 years old with ADHD.  -ADHD as diagnosed by standardized diagnostic criteria (such as DSM-IV, SDM-V, ICD-10) or other validated diagnostic tools.  -All genders, ethnicities, and severity of ADHD symptoms to be included. | -Adult without a definite ADHD diagnosis.  -Participants included not only adults with ADHD but also adults with other neuropsychological disorders(not co-morbidity). |
| Intervention | -Any non-pharmacotherapy aiming to improve the core symptoms of ADHD including Cognitive Behavioral Therapy (CBT), Neurofeedback therapy (NF)， noninvasive brain stimulation (NIBS) and so on. | -Any pharmacotherapy(such as methylphenidate, amphetamine, atomoxetine, guanfacine and so on)  The duration of the intervention was less than seven days. |
| Comparison | -Waiting list, usual care, active or semi-active control and equivalent control intervention.  -Studies will be retained if the comparator group is a different non-pharmacotherapy to facilitate direct comparisons in the network meta-analysis (e.g., CBT vs. PE). | -No suitable control or other non-pharmacotherapy comparator.  -The only comparator condition reported is a similar format to the non-pharmacotherapy group (e.g., comparison of two forms of the same non-pharmacotherapy, SCP-NF vs fNIRS-NF), which would not allow the  study to contribute data to a comparison (edge) in the network. |
| Outcome | -The change in the severity of ADHD core symptoms in both the short-term and long-term, as measured by clinician ratings, observer ratings, or self-assessment.  -The changes in co-occurring depression and anxiety, considering both short-term and long-term effects. | -Studies that did not report primary outcome measures (those with short-term effects but no follow-up results were retained).  -Studies reporting primary core symptoms using subscale results (e.g. inattention). |
| Study | -RCTs (individual design, cluster design, or the first half of crossover) | Non-RCTs |

## TABLE 6: Rating Scales Incorporated

| **Outcome** | **Abbreviations** | **Full Forms** |
| --- | --- | --- |
| ADHD core symptom | | |
|  | ADHD-RS | ADHD Rating Scale |
|  | ADHS-SB | German ADHD self-rating scale for symptoms in adulthood |
|  | AISRS | Adult ADHD Investigator Symptom Rating  Scale |
|  | ASRS | ADHD Self-Report Scale Symptom |
|  | ASRS v1.1 | ADHD Self-Report Scale Symptom 1.1 Version |
|  | ASRS-BPV | ASRS Brazilian Portuguese Version |
|  | BAARS-IV | Barkley Adult ADHD Rating Scale IV Version |
|  | BCS-S | The Barkley ADHD Current Symptoms Scale-Self report |
|  | CAARS-INV | Conners Adult ADHD Rating Scales Investigator Version |
|  | CAARS-K | Conners’ Adult ADHD Rating Scale-Korean |
|  | CAARS-O | Conners Adult ADHD Rating Scales–Observer Report |
|  | CAARS-S | Conners Adult ADHD Rating Scales–Self Report |
|  | CASRS | Clinician-administered version of the Adult ADHD  Self-report Scale |
|  | CSS | The Current Symptoms Scale Self-Report Form |
|  | DSM-IIIR-SC | DSM-IIIR Symptom Checklist |
|  | K-SADS | The Kiddie-Schedule for Affective Disorders and Schizophrenia (ADHD section) |
|  | AOS | Adult Organization Scale |
| Depression and Anxiety | | |
|  | BAI | Beck Anxiety Inventory |
|  | BDI | Beck Depression Inventory |
|  | BDI-2 | Beck Depression Inventory 2 Version |
|  | HADS | Hospital Anxiety and Depression |
|  | SAS | Self-rating Anxiety Scale |
|  | SDS | Self-rating Depression Scale |
|  | STAI | State-Trait Anxiety Inventory |
|  |  |  |

## TABLE 7: Definitions of Non-pharmacotherapies and Control Group/Condition

| **Type of Intervention** | **Abbreviation** | **Definition** |
| --- | --- | --- |
| Control Intervention | CON | Control intervention set up to evaluate the experimental group intervention including  (1) blank and wait-list control;  (2) drug placebo;  (3) treatment as usual (TAU);  (4) semi-active or active control, such as sham rTMS, brochure-assisted psychoeducation and so on. |
| Cognitive Behavioral Therapy | CBT | Cognitive Behavioral Therapies help participants become aware of inaccurate or negative thinking so they can view challenging situations more clearly and respond to them in a more effective way with or without TAU, including  (1) CBT;  (2) Internet-based CBT (iCBT);  (3) group CBT (gCBT);  (4) Mega Cognitive Therapy (MCT) [1];  (5) Dialectical Behavior Therapy (DBT)[2]. |
| Cognitive Therapy | CT | Treatment that directly focused on challenging cognitive skills such as attention, working memory and impulsivity[3,4], including  (1) Computerized Cognitive Training (CCT);  (2) Cognitive Remediation Program (CRP). |
| Mindfulness-Based Cognitive Therapy | MC | Mindfulness related interventions focus on the participants’ present-moment experiences and thoughts [5,6], including  (1) Mindfulness-Based Cognitive Therapy (MBCT);  (2) Mindfulness Awareness Practice (MAP); |
| Neurofeedback | NF | A kind of biofeedback, which teaches self-control of brain functions to subjects by measuring brain waves and providing a feedback signal [8], including  (1) Neurofeedback training;  (2) Slow Cortical Potential Neurofeedback (SCP-NF);  (3) Functional Near-infrared Spectroscopy Neurofeedback (fNIRS-NF) |
| Noninvasive Brain Stimulation | NIBS | A collection of technologies and techniques designed to modulate brain excitability through transcranial stimulation [10], including  (1) Transcranial Magnetic Stimulation  (2) Transcranial Direct Current Stimulation (tDCS) |
| Psychoeducation | PE | An approach that aims at improving the patients’ understanding and awareness of the disorder can offer insight into past difficulties and improve the patient’s general functioning [9]. |
| Self-Alert Training | SAT | An endogenous technique to teach participants to transiently increase their arousal at regular intervals in order to offset the periodic decreases in endogenous control that determine momentary lapses of attention [7]. |
| Taichi | Taichi | Tai Chi is an increasingly popular mind-body exercise that encourages mindful attention to the body in motion， which integrates low-impact flowing movements with a diverse set of cognitive skills relevant to ADHD[11]. |
| Vitamin–mineral treatment | VWT | A broad-based micronutrient treatment consisting mainly of vitamins and minerals, without omega fatty acids [12]. |
| Working Memory Training | WMT | A specific brain training method aimed at improving working memory deficits in ADHD[13]. |

**Reference**

[1] Schönenberg M, Wiedemann E, Schneidt A, Scheeff J, Logemann A, Keune PM, Hautzinger M. Neurofeedback, sham neurofeedback, and cognitive-behavioural group therapy in adults with attention-deficit hyperactivity disorder: a triple-blind, randomised, controlled trial. Lancet Psychiatry. 2017 Sep;4(9):673-684.

[2] O'connell, B., & Dowling, M. (2014). Dialectical behaviour therapy (DBT) in the treatment of borderline personality disorder. *Journal of psychiatric and mental health nursing*, *21*(6), 518-525.

[3] Stevenson, C. S., Whitmont, S., Bornholt, L., Livesey, D., & Stevenson, R. J. (2002). A cognitive remediation programme for adults with attention deficit hyperactivity disorder. Australian & New Zealand Journal of Psychiatry, 36(5), 610-616.

[4] Stern, A., Malik, E., Pollak, Y., Bonne, O., & Maeir, A. (2016). The efficacy of computerized cognitive training in adults with ADHD: A randomized controlled trial. Journal of attention disorders, 20(12), 991-1003.

[5] Creswell JD. Mindfulness Interventions. Annu Rev Psychol. 2017 Jan 3;68: 491-516.

[6] Poissant, H., Moreno, A., Potvin, S., & Mendrek, A. (2020). A meta-analysis of mindfulness-based interventions in adults with attention-deficit hyperactivity disorder: Impact on ADHD symptoms, depression, and executive functioning. Mindfulness, 11, 2669-2681.

[7] Salomone S, Fleming GR, Shanahan JM, Castorina M, Bramham J, O'Connell RG, Robertson IH. The effects of a Self-Alert Training (SAT) program in adults with ADHD. Front Hum Neurosci. 2015 Feb 10; 9:45.

[8] Marzbani H, Marateb HR, Mansourian M. Neurofeedback: A Comprehensive Review on System Design, Methodology and Clinical Applications. Basic Clin Neurosci. 2016 Apr;7(2):143-58.

[9] Bachmann K, Lam AP, Sörös P, Kanat M, Hoxhaj E, Matthies S, Feige B, Müller H, Özyurt J, Thiel CM, Philipsen A. Effects of mindfulness and psychoeducation on working memory in adult ADHD: A randomised, controlled fMRI study. Behav Res Ther. 2018 Jul; 106:47-56.

[10] Boes AD, Kelly MS, Trapp NT, Stern AP, Press DZ, Pascual-Leone A. Noninvasive Brain Stimulation: Challenges and Opportunities for a New Clinical Specialty. J Neuropsychiatry Clin Neurosci. 2018 Summer;30(3):173-179.

[11] Converse AK, Barrett BP, Chewning BA, Wayne PM. Tai Chi training for attention deficit hyperactivity disorder: A feasibility trial in college students. Complement Ther Med. 2020 Sep;53:102538.

[12] Rucklidge JJ, Johnstone J, Gorman B, Boggis A, Frampton CM. Moderators of treatment response in adults with ADHD treated with a vitamin-mineral supplement. Prog Neuropsychopharmacol Biol Psychiatry. 2014 Apr 3;50:163-71.

[13] Klingberg, T. (2010). Training and plasticity of working memory. Trends in cognitive sciences, 14(7), 317-324.

## TABLE 8: Characteristics of Studies Included

| **Study** | **Area** | **Diagnostic Criteria** | **Intervention** | **N** | **Male(%)** | **Age (M,SD)** | **Duration** | **Frequency** | **Follow-up** | **Primary Outcome** | **Secondary Outcome** |
| --- | --- | --- | --- | --- | --- | --- | --- | --- | --- | --- | --- |
| Alyagon2020 | Israel | DSM-IV | NIBS | 15 | 2 (13.33) | 26.62(0.66) | 3w | 5t/w | 4w | CAARS-S | BDI |
|  |  |  | CON | 14 | 3 (21.43) | 27.64(1.58) |  |  |  |  |  |
| Anastopoulos2021 | USA | DSM-V | CBT | 119 | 42 (35.29) | 19.7(2.2) | 8w | NA | 2-3w | CAARS-S | BDI, BAI |
|  |  |  | CON | 131 | 43 (13.33) | 19.6(2.1) |  |  |  |  |  |
| Anne2022 | Norway | DSM-IV | CBT | 60 | 28 (21.43) | 36.5(21~59) | 14w | 1t/w | 24w | ASRS | BDI, BAI |
|  |  |  | CON | 61 | 25 (35.29) | 37.5(21~57) |  |  |  |  |  |
| Bachmann2018 | Germany | DSM-IV | MC | 21 | 8 (32.82) | 40.26 (13.81) | 8w | 1t/w | NA | CAARS-O | NA |
|  |  |  | PE | 19 | 10 (46.67) | 40 (10.58) |  |  |  |  |  |
| Barth2021 | Germany | ADHS-SB | NF | 26 | 12 (40.98) | 33.62(10.24) | 6w | 1-5t/w | 24w | ADHS-SB | BDI |
|  |  |  | CON | 20 | 6 (38.1) | 33.65(12.64) |  |  |  |  |  |
| BleichCohen2021 | Israel | DSM-V | NIBS | 24 | 17 (52.63) | 36.5(8.7) | 3w | 5t/w | 4w | CAARS-S | BDI |
|  |  |  | CON | 16 | 8 (46.15) | 34.7(9.2) |  |  |  |  |  |
| Brynjar Emilsson2011 | UK | DSM-IV | CBT | 26 | NA | NA | 7.5w | 2t/w | 12w | BCS-S | BDI, BAI |
|  |  |  | CON | 25 | NA | NA |  |  |  |  |  |
| Cachoeira2016 | Brazil | DSM-V | NIBS | 8 | 4 (50) | 33.75(3.65) | 5d | 7t/w | 4w | ASRS-v1.1 | NA |
|  |  |  | CON | 9 | 4 | 31(6.17) |  |  |  |  |  |
| Caroline2002 | Australia | SSIAA, DSM-IIIR symptom checklist | CT | 22 | 16 | 36.4(10) | 8w | 1t/w | 8w | DSM-IIIR SC | NA |
|  |  |  | CON | 21 | 13 (50) | 35.3(8.8) |  |  |  |  |  |
| Converse2020 | USA | NA | Taichi | 9 | (44.44) | NA | 7w | 2t/w | NA | CAARS-S | NA |
|  |  |  | CON | 5 | (72.73) | NA |  |  |  |  |  |
| Cowley2016 | Finland | ASRS-v1.1 | NF | 25 | 11 (61.9) | 35.72(9.66) | 8-16w | 2-4t/w | NA | ASRS-v1.1 | NA |
|  |  |  | CON | 29 | 14 | 36.45(10.86) |  |  |  |  |  |
| Dentz2020 | Canada | DSM-IV | WMT | 23 | 7 | 39.48(2.64) | 5w | 5t/w | 2w | CAARS-S | NA |
|  |  |  | CON | 21 | 10 (44) | 43.90(11.8) |  |  |  |  |  |
| Dittner2018 | UK | DSM-IV | CBT | 30 | 23 (48.28) | 35.7(9) | 30w | 2t/w | 12w | CSS | HADS |
|  |  |  | CON | 30 | 18 (30.43) | 36.1(10.4) |  |  |  |  |  |
| Fleming2019 | USA | DSM-V | CBT | 17 | 10 (47.62) | 21.2(1.67) | 8w | 1t/w | 12w | BAARS-IV | BDI, BAI |
|  |  |  | CON | 16 | 9 (76.67) | 21.5(1.12) |  |  |  |  |  |
| Gu2018 | China | DSM-V | MC | 28 | 16 (60) | 20.21(1.03) | 8w | 1t/w | 12w | CAARS-S | BDI-2, BAI |
|  |  |  | CON | 26 | 14 (58.82) | 20.38(1.02) |  |  |  |  |  |
| Hepark2019 | The Netherlands | DSM-IV | MC | 55 | 34 (56.25) | 36.5(10) | 8-12w | 1t/w | NA | CAARS-INV | BDI-2, STAI |
|  |  |  | CON | 48 | 22 (57.14) | 36.2(9) |  |  |  |  |  |
| Hoxhaj2018 | Germany | DSM-IV | MC | 41 | 18 (53.85) | 40.51(9.48) | 8w | 1t/w | 32w | CAARS-O | BDI |
|  |  |  | PE | 40 | 21 (61.82) | 38.50(11.83) |  |  |  |  |  |
| Jang2021 | Korea | ASRS-v1.1 | CBT | 23 | 10 (45.83) | 26.7(8.97) | 4w | NA | NA | CAARS-S | SAS |
|  |  |  | CON | 23 | 10 (43.9) | 22.87(5.44) |  |  |  |  |  |
| Janssen2018 | The Netherlands | DSM-IV | MC | 60 | 28 (52.5) | 39.7(11.1) | 8w | 1t/w | 12w | CAARS-INV | NA |
|  |  |  | CON | 60 | 28 (43.48) | 39.0(10.1) |  |  |  |  |  |
| Leffa2022 | Brazil | DSM-V | NIBS | 32 | 13 (43.48) | 38.2(10.3) | 4w | 7t/w | NA | CASRS | BDI, BAI |
|  |  |  | CON | 32 | 21 (46.67) | 38.4(9.1) |  |  |  |  |  |
| Moritz2020 | Brazil | DSM-V | CBT | 16 | (46.67) | NA | 12w | 1t/w | 4w | ASRS-BPV | NA |
|  |  |  | CON | 15 | (40.63) | NA |  |  |  |  |  |
| Nakashima2022 | Japan | DSM-IV | CBT | 24 | 3 (65.63) | 39.11(9.62) | 8w | 1t/w | 8w | CAARS-O | NA |
|  |  |  | CON | 24 | 3 | 39.67(9.79) |  |  |  |  |  |
| Nasri2023 | China | DSM-IV | CBT | 36 | 11 | 36.7(11.4) | 12w | NA | 12w | ASRS | NA |
|  |  |  | CON | 31 | 14 (12.5) | 37.2(10.3) |  |  |  |  |  |
| Pan2022 | Israel | DSM-IV | CBT | 49 | 27 (12.5) | 26.84(5.65) | NA | NA | 12w | ADHD-RS | SAS, SDS |
|  |  |  | CON | 49 | 31 (30.56) | 24.78(5.59) |  |  |  |  |  |
| Paz2018 | Sweden | DSM-V | NIBS | 9 | 6 (45.16) | 32.11(6.47) | 4w | 1t/w | NA | CAARS-S | NA |
|  |  |  | CON | 13 | 8 (55.1) | 30.85(6.82) |  |  |  |  |  |
| Pettersson2017 | UK | DSM-IV | CBT | 14 | 6 (63.27) | 39.64(12.44) | 10w | 1t/w | 24w | CSS | BDI, BAI |
|  |  |  | CON | 18 | 4 (66.67) | 33.78(10.07) |  |  |  |  |  |
| Rucklidge2014 | Korea | DSM-IV、 K-SADS-PL | VMT | 42 | 22 (61.54) | 36.39(14.2) | 8w | 7t/w | NA | CAARS-O | NA |
|  |  |  | CON | 38 | 31 (42.86) | 33.95(12.26) |  |  |  |  |  |
| Ryoo2015 | America | CAARS-K | NF | 8 | (22.22) | NA | 5w | 3t/w | 4w | CAARS-K | NA |
|  |  |  | CON | 8 | (52.38) | NA |  |  |  |  |  |
| Safren2005 | America | DSM-IV | CBT | 16 | (81.58) | NA | 15w | NA | NA | ADHD-RS | BDI, BAI |
|  |  |  | CON | 15 | NA | NA |  |  |  |  |  |
| Safren2010 | USA | DSM-IV | CBT | 43 | 24 | 42.3(10.3) | 15w | NA | 12w | ADHD-RS | NA |
|  |  |  | CON | 43 | 24 | 44(12.2) |  |  |  |  |  |
| Salomone2015 | Germany | DSM-IV | SAT | 24 | 16 | 32.7(12.4) | 5w | 5t/w | 12w | CAARS-S | BDI, BAI |
|  |  |  | CON | 27 | 20 (55.81) | 31.6(11.3) |  |  |  |  |  |
| Schönenberg2017 | America | DSM-IV | CBT | 37 | 19 (55.81) | 33.97(11.21) | 15w | 1t/w | 24w | CAARS-S | BDI, STAI |
|  |  |  | NF | 38 | 23 (66.67) | 38.21(11.31) |  |  |  |  |  |
| Solanto2009 | Israel | DSM-IV | CBT | 45 | 13 (74.07) | 41.04(11.59) | 12w | NA | NA | AISRS | BDI |
|  |  |  | CON | 43 | 17 (51.35) | 42.37(12.09) |  |  |  |  |  |
| Stern2016 | Spain | DSM-IV | CT | 34 | 15 (60.53) | 37.99(10.36) | 12w | 5t/w | NA | ASRS-v1.1 | NA |
|  |  |  | CON | 26 | 11 (28.89) | 36.41(9.90) |  |  |  |  |  |
| Vidal2013 | Canada | DSM-IV | CBT | 17 | 11 (39.53) | 39.53(5.91) | 12w | 1t/w | NA | ADHD-RS | BDI |
|  |  |  | PE | 15 | 6 (44.12) | 39.4(9.3) |  |  |  |  |  |
| Woltering2019 | UK | NA | WMT | 29 | 16 (42.31) | 24.3(3.4) | 5w | 5t/w | NA | ASRS-v1.1 | NA |
|  |  |  | CON | 28 | 15 (64.71) | 23.5(3.4) |  |  |  |  |  |
| Young2015 | Sweden | DSM-IV | CBT | 48 | 18 (40) | 34.19(10.58) | 7.5w | 2t/w | 12w | K-SADS | BDI, BAI |
|  |  |  | CON | 57 | 15 (55.17) | 36.17(12.75) |  |  |  |  |  |

## TABLE 9: The League Table of Depression

| **Depression** | Comparison of treatments: Standardized Mean different(95%confidence intervals)/ Effect of intervention in each row compared with intervention in each column | | | | | |
| --- | --- | --- | --- | --- | --- | --- |
| **CBT** | NA | -3.69 ( -8.45, 1.07) | NA | 3.23 ( -1.59, 8.06) | NA | **-4.12 ( -5.52,-2.72)** |
| **-3.36 ( -6.56,-0.16)** | **MC** | NA | NA | **8.70 ( 3.77, 13.63)** | NA | -1.30 ( -4.64, 2.04) |
| **-4.24 ( -7.66,-0.81)** | -0.88 ( -5.40, 3.64) | **NF** | NA | NA | NA | 0.62 ( -4.11, 5.35) |
| **-4.23 ( -7.84,-0.62)** | -0.87 ( -5.38, 3.63) | 0.01 ( -4.78, 4.79) | **NIBS** | NA | NA | 0.07 ( -3.27, 3.42) |
| **4.26 ( 0.48, 8.05)** | **7.62 ( 3.81, 11.44)** | **8.50 ( 3.47, 13.53)** | **8.50 ( 3.41,13.59)** | **PE** | NA | NA |
| -4.78 ( -9.71, 0.15) | -1.42 ( -7.04, 4.20) | -0.54 ( -6.39, 5.30) | -0.55 ( -6.35, 5.25) | **-9.05 (-15.14, -2.95)** | **SAT** | 0.62 ( -4.12, 5.36) |
| **-4.16 ( -5.51,-2.81)** | -0.80 ( -3.82, 2.22) | 0.08 ( -3.34, 3.50) | 0.07 ( -3.27, 3.42) | **-8.42 (-12.26, -4.59)** | 0.62 ( -4.12, 5.36) | **CON** |

Data are standardized mean difference (95%CI) between therapies. Results in bold are significant. Negative values favour the therapy in the row and positive values favour the therapy in the column. Non-pharmacological therapies are reported in alphabetical order. The top section of the figure displays the results of direct comparisons, while the bottom section shows the results of mixed comparisons. *CBT:* Cognitive Behavioral Therapy; *CON:* Control; *MC:* Mindfulness-based Cognitive Therapy; *NF:* Neurofeedback; *NIBS:* Noninvasive Brain Stimulation; *PE:* Psychoeducation; *SAT:* Self-Alert Training. *NA*: No available data.

| **FU of Depression** | Comparison of treatments: Standardized Mean different(95%confidence intervals)/ Effect of intervention in each row compared with intervention in each column | | | | | |
| --- | --- | --- | --- | --- | --- | --- |
| **CBT** | NA | **-11.25 (-17.12,-5.39)** | NA | NA | NA | **-3.40 ( -5.53,-1.27)** |
| -3.17 ( -9.11, 2.77) | **MC** | NA | NA | 2.88 ( -2.71, 8.46) | NA | -0.72 ( -6.29, 4.85) |
| **-7.59 (-11.77,-3.40)** | -4.41 (-11.36, 2.54) | **NF** | NA | NA | NA | 0.38 ( -5.19, 5.96) |
| -1.72 ( -7.68, 4.25) | 1.46 ( -6.44, 9.35) | 5.87 ( -1.11,12.84) | **NIBS** | NA | NA | -2.17 ( -7.77, 3.43) |
| -0.30 ( -8.45, 7.86) | 2.88 ( -2.71, 8.46) | 7.29 ( -1.63,16.21) | 1.42 ( -8.25,11.10) | **PE** | NA | NA |
| -3.16 ( -9.12, 2.80) | 0.01 ( -7.88, 7.91) | 4.43 ( -2.54,11.40) | -1.44 ( -9.36, 6.47) | -2.86 (-12.53, 6.81) | **SAT** | -0.73 ( -6.32, 4.86) |
| **-3.89 ( -5.95,-1.83)** | -0.72 ( -6.29, 4.85) | 3.70 ( -0.46, 7.85) | -2.17 ( -7.77, 3.43) | -3.59 (-11.48, 4.30) | -0.73 ( -6.32, 4.86) | **CON** |

## TABLE 10: The League Table of Follow-up of Depression

Data are standardized mean difference (95%CI) between therapies. Results in bold are significant. Negative values favour the therapy in the row and positive values favour the therapy in the column. Non-pharmacological therapies are reported in alphabetical order. The top section of the figure displays the results of direct comparisons, while the bottom section shows the results of mixed comparisons. *CBT:* Cognitive Behavioral Therapy; *CON:* Control; *MC:* Mindfulness-based Cognitive Therapy; *NF:* Neurofeedback; *NIBS:* Noninvasive Brain Stimulation; *PE:* Psychoeducation; *SAT:* Self-Alert Training. *NA*: No available data.

## TABLE 11: The League Table of Anxiety

| **Anxiety** | Comparison of treatments: Standardized Mean different(95%confidence intervals)/ Effect of intervention in each row compared with intervention in each column | | | | |
| --- | --- | --- | --- | --- | --- |
| **CBT** | NA | -0.46 (-3.66, 2.73) | NA | NA | **-2.12 (-3.18,-1.07)** |
| -0.60 (-3.11, 1.91) | **MC** | NA | NA | NA | -1.52 (-3.80, 0.75) |
| -0.46 (-3.66, 2.73) | 0.14 (-3.92, 4.20) | **NF** | NA | NA | NA |
| -3.08 (-6.46, 0.30) | -2.48 (-6.42, 1.45) | -2.62 (-7.27, 2.03) | **NIBS** | NA | 0.96 (-2.25, 4.17) |
| -1.79 (-5.19, 1.60) | -1.19 (-5.14, 2.75) | -1.33 (-5.99, 3.33) | 1.29 (-3.26, 5.84) | **SAT** | -0.33 (-3.56, 2.90) |
| **-2.12 (-3.18,-1.07)** | -1.52 (-3.80, 0.75) | -1.66 (-5.02, 1.71) | 0.96 (-2.25, 4.17) | -0.33 (-3.56, 2.90) | **CON** |

Data are standardized mean difference (95%CI) between therapies. Results in bold are significant. Negative values favour the therapy in the row and positive values favour the therapy in the column. Non-pharmacological therapies are reported in alphabetical order. The top section of the figure displays the results of direct comparisons, while the bottom section shows the results of mixed comparisons. *CBT:* Cognitive Behavioral Therapy; *CON:* Control; *MC:* Mindfulness-based Cognitive Therapy; *NF:* Neurofeedback; *NIBS:* Noninvasive Brain Stimulation; *SAT:* Self-Alert Training. NA: No available data.

| **FU of Anxiety** | Comparison of treatments: Standardized Mean different(95%confidence intervals)/ Effect of intervention in each row compared with intervention in each column | | | |
| --- | --- | --- | --- | --- |
| **CBT** | NA | **-7.25 (-10.57,-3.94)** | NA | **-3.38 ( -4.76,-2.00)** |
| -1.15 ( -4.58, 2.27) | **MC** | NA | NA | -2.23 ( -5.36, 0.90) |
| **-7.25 (-10.57,-3.94)** | **-6.10 (-10.86,-1.34)** | **NF** | NA | NA |
| -3.36 ( -6.79, 0.08) | -2.20 ( -6.64, 2.23) | 3.90 ( -0.87, 8.67) | **SAT** | -0.03 ( -3.17, 3.11) |
| **-3.38 ( -4.76,-2.00)** | -2.23 ( -5.36, 0.90) | **3.87 ( 0.28, 7.46)** | -0.03 ( -3.17, 3.11) | **CON** |

## TABLE 12: The League Table of the Follow-up of Anxiety

Data are standardized mean difference (95%CI) between therapies. Results in bold are significant. Negative values favour the therapy in the row and positive values favour the therapy in the column. Non-pharmacological therapies are reported in alphabetical order. The top section of the figure displays the results of direct comparisons, while the bottom section shows the results of mixed comparisons. *CBT:* Cognitive Behavioral Therapy; *CON:* Control; *MC:* Mindfulness-based Cognitive Therapy; *NF:* Neurofeedback; *SAT:* Self-Alert Training. NA: No available data.

## TABLE 13: Evaluation of heterogeneity

We use the tau square (τ^2^) test and p-value to qualitatively analyze the statistical heterogeneity between the studies. The larger the τ^2^ and the smaller the p-value, the greater the possibility of heterogeneity, on the contrary, the smaller the existence of heterogeneity. In addition, I^2^ is a parameter for quantitative analysis of the heterogeneity between the results of each study. Its value is distributed from 0-100%. When I^2^ is less than 25%, it means that the heterogeneity is low,25%-50% means that the heterogeneity is moderate, I^2^ > 75% means high heterogeneity. In summary, when I^2^ > 50%, it means that there is substantial heterogeneity.

| Primary outcome | τ2 | Q | df | P | I^2^ | Heterogeneity assessment |
| --- | --- | --- | --- | --- | --- | --- |
| Core Symptom | 4.390 | 722.14 | 27 | 0.001 | 96.3 | High |
| Depression | 5.737 | 670.38 | 15 | 0.001 | 97.8 | High |
| Anxiety | 2.602 | 280.56 | 10 | 0.001 | 96.4 | High |
| FU of Core Symptom | 2.809 | 202.67 | 14 | 0.001 | 93.1 | High |
| FU of Depression | 7.995 | 315.67 | 7 | 0.001 | 97.8 | High |
| FU of Anxiety | 2.428 | 94.66 | 5 | 0.001 | 94.9 | High |

## TABLE 14: Evaluation of inconsistency

| Outcomes |  | SIDE splitting | | the Design-by-Treatment test | | | |
| --- | --- | --- | --- | --- | --- | --- | --- |
|  | Number  of studies | Number of inconsistent  comparisons out of total | Percentage of inconsistent  comparisons out of total | Q | df | τ2 | p-value |
| ADHD symptom | 37 | 0 | 0 | 2.72 | 2 | 4.331 | 0.257 |
| Depression | 21 | 0 | 0 | 0.52 | 2 | 6.077 | 0.773 |
| Anxiety | 15 | 0 | 0 | 0 | 0 | 2.602 | -- |
| FU of ADHD symptom | 20 | 0 | 0 | 0.35 | 1 | 2.664 | 0.555 |
| FU of Depression | 13 | 0 | 0 | 3.61 | 1 | 6.725 | 0.058 |
| FU of Anxiety | 9 | 0 | 0 | 0 | 0 | 2.428 | -- |

| Covariate | Shared beta ( median and 95%Crl ) | | | | | |
| --- | --- | --- | --- | --- | --- | --- |
|  | Core Symptom | Depression | Anxiety | FU of Core Symptom | FU of Depression | FU of Anxiety |
| Publish year | 0.101 (-3.041, 3.538) | **4.758 (0.503, 8.738)** | **1.107 (5.294, 8.809)** | -0.393 (-2.922, 1.702) | 3.121 (-4.167, 9.499) | 3.280 (-89.966, 44.140) |
| Area | 0.751 (-2.346, 4.602) | 2.451 (-2.763, 7.553) | -0.951 (-69.447, 9.003) | -1.697 (-4.595, 0.596) | 2.408 (-5.999, 11.391) | 2.033 (-46.865, 59.919) |
| Mean Age | -2.164 (-5.220, 27.396) | 0.017 (-8.643, 5.364) | 1.283 (-4.085, 7.425) | -0.068 (-2.363, 2.150) | 4.230 (-1.082, 10.164) | -3.552 (-46.962, 32.860) |
| Sample Size | 0.373 (-6.135, 39.875) | 2.082 (-2.225, 6.524) | 1.669 (-5.601, 40.311) | 0.607 (-1.895, 2.889) | 0.325 (-7.126, 5.706) | -13.777(-103.903,116.550) |
| Percentage of Male | -1.648 (-5.341, 1.431) | 1.316 (-4.913, 7.805) | **7.763 (2.483, 18.155)** | 0.657 (-1.973, 3.520) | -1.077 (-8.938, 6.672) | -5.226 (-111.011, 25.970) |
| Diagnosis method | -0.506 (-3.874, 3.450) | -1.018 (-35.732, 5.225) | -2.180 (-7.746, 11.941) | 0.153 (-2.549, 3.399) | 1.903 (-6.803, 10.060) | -20.948 (-77.267, 33.180) |
| Scale Type | 2.169 (-1.083, 6.172) | -3.158 (-8.399, 11.786) | 3.038 (-3.328, 38.935) | 0.223 (-2.281, 2.954) | -3.016 (-8.787, 3.081) | -12.179 (-127.705, 69.410) |
| Scale Overall Score | **4.140 (0.383, 8.163)** | -- | -- | 2.400 (-0.351, 5.285) | -- | -- |
| Intervention Duration | -0.225 (-4.085, 3.724) | 1.916 (-3.083, 7.543) | **8.201 (4.362, 12.806)** | -0.117 (-2.945, 2.799) | -0.551 (-7.145, 6.623) | **23.829 (17.990, 29.998)** |
| Intervention Frequency | 1.474 (-6.956, 7.130) | 0.756 (-9.619, 10.472) | 10.585(-12.801,125.053) | 5.137 (-2.588, 14.739) | **18.050 (6.372, 26.543)** | 24.840 (-119.890,115.970) |
| Follow-up Length | -- | -- | -- | -3.062 (-6.198, 0.044) | 4.891 (-5.497, 10.691) | **63.912 (13.310, 98.411)** |

## TABLE 15: Outcome of Network Meta-Regression

## TABLE 16: Subgroup Analysis

| **Outcomes (original results)** | | | **Subgroup** | | | **Subgroup** | | |
| --- | --- | --- | --- | --- | --- | --- | --- | --- |
| Core symptom | | | Scale Overall Score < 66 | | | Scale Overall Score ≥ 66 | | |
| Contrast to CON | SMD | 95%Crl | Contrast to CON | SMD | 95%Crl | Contrast to CON | SMD | 95%Crl |
| PE | −6.38 | **[−9.25, −3.52]** | PE | −4.28 | **[−6.57, −1.98]** | NIBS | −12.71 | **[−23.10, −2.33]** |
| MC | −5.07 | **[−7.29, −2.84]** | CBT | −3.53 | **[−4.39, −2.68]** | MC | −7.21 | **[−13.74, −0.67]** |
| CBT | −4.43 | **[−5.50, −3.37]** | VMT | −3.02 | **[−5.91, −0.13]** | CBT | −6.89 | **[−11.72, −2.06]** |
| CT | −4.02 | **[−7.05, −0.99]** | SAT | −3.00 | **[−5.98, −0.03]** | NF | −2.43 | [ −8.04, 3.18] |
| VMT | −3.02 | [−7.18, 1.14] | WMT | −2.77 | [−5.71, 0.17] | CT | −1.56 | [−10.73, 7.61] |
| SAT | −3.00 | [−7.22, 1.22] | MC | −2.37 | **[−4.63, −0.11]** | WMT | −0.00 | [ −9.16, 9.16] |
| NIBS | −2.38 | **[−4.37, −0.39]** | Taichi | −2.20 | [−5.33, 0.93] |  |  |  |
| Taichi | −2.20 | [−6.53, 2.13] | NIBS | −1.29 | [−2.74, 0.17] |  |  |  |
| WMT | −1.37 | [−4.31, 1.58] | NF | 2.39 | [−0.53, 5.31] |  |  |  |
| NF | −0.28 | [−2.47, 1.91] |  |  |  |  |  |  |
| Depression | | | Publish Year < 2017 | | | Publish Year ≥ 2017 | | |
| PE | −8.42 | **[−12.26, −4.59]** | PE | −4.38 | **[−7.43, −1.33]** | PE | −10.00 | **[−16.49, −3.51]** |
| CBT | −4.16 | **[ −5.51, −2.81]** | CBT | −1.15 | [−2.45, 0.16] | CBT | −5.62 | **[ −7.46, −3.79]** |
| MC | −0.80 | [ −3.82, 2.22] | SAT | 0.62 | [−1.99, 3.23] | MC | −1.30 | [ −4.95, 2.36] |
| NIBS | 0.07 | [ −3.27, 3.42] |  |  |  | NF | −0.65 | [ −4.43, 3.13] |
| NF | 0.08 | [ −3.34, 3.50] |  |  |  | NIBS | 0.07 | [ −3.59, 3.74] |
| SAT | 0.62 | [ −4.12, 5.36] |  |  |  |  |  |  |
| Anxiety | | | Publish Year < 2017 | | | Publish Year ≥ 2017 | | |
| CBT | −2.12 | **[−3.18, −1.07]** | CBT | −1.35 | **[−1.71, −0.99]** | CBT | −2.73 | **[−4.24, −1.22]** |
| NF | −1.66 | [−5.02, 1.71] | SAT | −0.33 | [−0.98, 0.32] | NF | −2.26 | [−6.37, 1.84] |
| MC | −1.52 | [−3.80, 0.75] |  |  |  | MC | −1.53 | [−4.24, 1.19] |
| SAT | −0.33 | [−3.56, 2.90] |  |  |  | NIBS | 0.96 | [−2.87, 4.79] |
| NIBS | 0.96 | [−2.25, 4.17] |  |  |  |  |  |  |
| Anxiety | | | Percentage of Male < 54 (%) | | | Percentage of Male ≥ 54 (%) | | |
| CBT | −2.12 | **[−3.18, −1.07]** | CBT | −0.34 | [−1.66, 0.99] | CBT | −5.23 | **[−7.38, −3.08]** |
| NF | −1.66 | [−5.02, 1.71] | NIBS | 0.96 | [−1.69, 3.61] | NF | −4.76 | **[−9.29, −0.24]** |
| MC | −1.52 | [−3.80, 0.75] |  |  |  | MC | −1.53 | [−4.36, 1.31] |
| SAT | −0.33 | [−3.56, 2.90] |  |  |  | SAT | −0.33 | [−4.34, 3.69] |
| NIBS | 0.96 | [−2.25, 4.17] |  |  |  |  |  |  |
| Follow-up of Depression | | | Intervention Frequency < 2 (t/w) | | | Intervention Frequency ≥ 2 (t/w) | | |
| CBT | −3.89 | **[ −5.95, −1.83]** | PE | −3.59 | **[−4.50, −2.69]** | CBT | −2.18 | **[−2.68, −1.67]** |
| PE | −3.59 | [−11.48, 4.30] | MC | −0.72 | **[−1.27, −0.16]** | NIBS | −2.17 | **[−3.07, −1.27]** |
| NIBS | −2.17 | [ −7.77, 3.43] | CBT | 2.62 | [ 1.65, 3.59] | SAT | −0.73 | [−1.59, 0.13] |
| SAT | −0.73 | [ −6.32, 4.86] | NF | 13.87 | [11.72, 16.02] | NF | 0.38 | [−0.34, 1.10] |
| MC | −0.72 | [ −6.29, 4.85] |  |  |  |  |  |  |
| NF | 3.70 | [ −0.46, 7.85] |  |  |  |  |  |  |
| Follow-up of Anxiety | | | Intervention Duration < 11 (weeks) | | | Intervention Duration ≥ 11 (weeks) | | |
| CBT | −3.38 | **[−4.76, −2.00]** | MC | −2.23 | **[−2.98, −1.48]** | CBT | −36.37 | **[−44.14, −28.59]** |
| MC | −2.23 | [−5.36, 0.90] | CBT | −2.13 | **[−2.47, −1.79]** | NF | −29.11 | **[−36.99, −21.23]** |
| SAT | −0.03 | [−3.17, 3.11] | SAT | −0.03 | [−0.81, 0.76] |  |  |  |
| NF | 3.87 | **[ 0.28, 7.46]** |  |  |  |  |  |  |
| Follow-up of Anxiety | | | Follow-up Length < 12 (weeks) | | | Follow-up Length ≥ 12 (weeks) | | |
| CBT | −3.38 | **[−4.76, −2.00]** | CBT | −4.36 | **[−6.44, −2.28]** | CBT | −2.30 | **[−2.66, −1.94]** |
| MC | −2.23 | [−5.36, 0.90] | MC | −2.23 | [−6.48, 2.03] |  |  |  |
| SAT | −0.03 | [−3.17, 3.11] | SAT | −0.03 | [−4.29, 4.24] |  |  |  |
| NF | 3.87 | [ 0.28, 7.46] | NF | 2.89 | [−1.97, 7.75] |  |  |  |

## TABLE 17: The Risk of Bias Assessment for The Individual Included Studies

| Author, year | Randomization process | Deviations from intended interventions | Missing outcome data | Measurement of the outcome | Selection of the reported result | Overall |
| --- | --- | --- | --- | --- | --- | --- |
| Alyagon, 2020 | + | ? | + | + | ? | ? |
| Anastopoulos, 2021 | ? | - | + | - | ? | - |
| Anne, 2022 | + | - | + | - | + | - |
| Bachmann, 2018 | + | + | + | + | + | + |
| Barth, 2021 | + | - | + | - | + | - |
| Bleich Cohen, 2021 | + | ? | + | + | + | ? |
| Brynjar Emilsson,2011 | + | + | + | + | + | + |
| Cachoeira, 2016 | + | + | + | + | + | + |
| Caroline, 2002 | ? | + | + | - | ? | - |
| Converse, 2020 | ? | ? | + | - | - | - |
| Cowley, 2016 | ? | - | + | ? | - | - |
| Dentz, 2020 | ? | ? | + | + | ? | ? |
| Dittner, 2018 | + | + | + | - | + | - |
| Fleming, 2019 | ? | + | + | - | ? | - |
| Gu, 2018 | ? | + | + | + | ? | ? |
| Hepark, 2019 | ? | + | + | + | ? | ? |
| Hoxhaj, 2018 | + | - | + | + | ? | - |
| Jang, 2021 | ? | + | + | - | ? | - |
| Janssen, 2018 | + | + | + | + | + | + |
| Leffa, 2022 | + | + | + | + | + | + |
| Moritz, 2020 | + | ? | + | - | ? | - |
| Nakashima, 2022 | + | + | + | - | + | - |
| Nasri, 2023 | ? | + | + | + | + | ? |
| Pan, 2022 | + | + | + | - | + | - |
| Paz, 2018 | + | ? | + | + | + | ? |
| Pettersson, 2017 | + | + | + | - | ? | - |
| Rucklidge, 2014 | + | + | + | + | + | + |
| Ryoo, 2015 | - | + | + | - | ? | - |
| Safren, 2005 | ? | + | + | + | ? | ? |
| Safren, 2010 | + | + | + | + | + | + |
| Salomone, 2015 | + | + | ? | + | ? | ? |
| Schönenberg, 2017 | + | + | + | + | + | + |
| Solanto, 2009 | ? | + | + | + | ? | ? |
| Stern, 2016 | + | - | + | + | + | - |
| Vidal, 2013 | + | + | + | - | ? | - |
| Woltering, 2019 | ? | ? | + | - | + | - |
| Young, 2015 | + | + | + | + | + | + |

## TABLE 18: The Overall Certainty of Evidence (CINeMA)

| **Comparison** | **Number of studies** | **Within-study bias** | **Reporting bias** | **Indirectness** | **Imprecision** | **Heterogeneity** | **Incoherence** | **Confidence rating** | **Reason(s) for downgrading** |
| --- | --- | --- | --- | --- | --- | --- | --- | --- | --- |
| Core Symptom | | | | | | | | | |
| CBT: CON | 15 | Major concerns | Low risk | No concerns | Some concerns | Some concerns | Major concerns | Very low | [Within-study bias] |
| CBT:NF | 1 | Major concerns | Low risk | No concerns | No concerns | Major concerns | Major concerns | Very low | [Within-study bias] |
| CON:NF | 3 | Major concerns | Low risk | No concerns | Some concerns | Some concerns | Major concerns | Very low | [Within-study bias] |
| CON: NIBS | 5 | No concerns | Low risk | No concerns | Major concerns | No concerns | Major concerns | Very low | [Imprecision] |
| CON: WMT | 2 | Some concerns | Low risk | No concerns | No concerns | Major concerns | Major concerns | Very low | [Heterogeneity] |
| Depression | | | | | | | | | |
| CBT: CON | 12 | Some concerns | Low risk | No concerns | No concerns | Major concerns | No concerns | Very low | [Heterogeneity] |
| CBT:NF | 1 | Some concerns | Low risk | No concerns | No concerns | Major concerns | No concerns | Very low | [Heterogeneity] |
| CON:MC | 2 | Some concerns | Low risk | No concerns | Major concerns | No concerns | No concerns | Very low | [Imprecision] |
| CON:NF | 1 | Some concerns | Low risk | No concerns | Major concerns | No concerns | No concerns | Very low | [Imprecision] |
| CON: NIBS | 2 | No concerns | Low risk | No concerns | Major concerns | No concerns | No concerns | Low | [Imprecision] |
| CON: SAT | 1 | Some concerns | Low risk | No concerns | Major concerns | No concerns | No concerns | Very low | [Imprecision] |
| FU of Core Symptom | | | | | | | | | |
| CBT: CON | 10 | No concerns | Low risk | No concerns | No concerns | Major concerns | Major concerns | Very low | [Heterogeneity] |
| CON: NIBS | 3 | Some concerns | Low risk | No concerns | Major concerns | No concerns | Major concerns | Very low | [Imprecision] |

**Reason for Downgrading**

Based on the recommendations of the CINeMA online document (https://cinema.ispm.unibe.ch/), we only graded the results of ADHD core symptoms, depression and follow-up of core symptoms whether each module needs to be downgraded according to the following criteria. We referenced the CINeMA guideline (1) and a recent component NMA (2).

***Within-study bias***

Based on the risk of bias (Supplementary Table 17) for each domain, each study can be judged to have an overall “Low risk” or “High risk” of bias, or “Some concerns”. We selected the rule “Average RoB” (“Average RoB” uses a weighted average score for each relative effect estimate according to the percentage contribution of studies at each bias level). Based on these scores, estimates from each pairwise comparison were assigned a within-study bias of “No concerns”, “Some concerns”, or “Major concerns”.

***Across-study bias (publication bias)***

Our search was relatively comprehensive. Due to the language is not limited, Chinese studies were also included in the scope of our inclusion. Even if it is possible that we missed other small unpublished experiments, then it does not seem to affect our results. In Supplementary Figures 12-17, we evaluated the outcome of publication bias, and most of the comparison-adjusted funnel plots for non-pharmacological therapies showed no evidence of asymmetry. Based on the results of the funnel plots, we downgraded the corresponding comparisons.

***Indirectness***

Indirectness refers to the relevance of included studies to the research question. The study population, interventions, outcomes, and settings may not represent the context, population, or outcomes that reviewers aim to infer. We address these problems by limiting the included studies to adults with ADHD according to inclusion and exclusion criteria. Therefore, we did not downgrade for indirectness.

***Imprecision***

The outcome (core symptom, depression and FU of core symptom) of this network meta-analysis is a continuous variable, and the effect size measure for continuous outcomes chooses the standardized mean difference (SMD) of the change score (end-point minus baseline score) because the studies use different rating scales or units. We considered a clinically meaningful threshold for non-pharmacological therapies to be -0.2 or 0.2 (2). If the confidence interval crosses the line of no-effect and extends to values that favour the opposite intervention to that favoured by the point estimate, “Major concerns” is assigned. If only the null effect is included in the confidence intervals (and potentially also the clinically important value that favours the same intervention as the point estimate), “Some concerns” is assigned. Finally, “No concerns” is assigned to confidence intervals that only include the clinically important value that favours the same intervention as the point estimate. If the confidence interval lies entirely between the two clinically important values, “No concerns” is assigned.

***Heterogeneity***

For heterogeneity assessment, we used the same threshold as the clinically significant threshold mentioned above. Following CINeMA's automated recommendations, we based our conclusions on the agreement between confidence and prediction intervals. Specifically, CINeMA judged the agreement of these intervals in relation to the null effect and the clinically important effect opposite to the point estimate.

***Incoherence***

For incoherence assessment, we used CINeMA to compare direct and indirect evidence, incorporating results from the design-by-treatment interaction test and Separating Indirect from Direct Evidence (SIDE). When effect estimates are based on both direct and indirect evidence, a SIDE p-value > 0.10 indicates “No concerns”. If the p-value is < 0.10, CINeMA assigns the risk level based on the agreement between direct and indirect estimates, using their 95% confidence intervals within the clinically important effect range. For estimates based solely on direct or indirect evidence, the judgment relies on the p-value of the design-by-treatment interaction test. If this test is not estimable due to the absence of closed evidence loops, “Major concerns” are assigned to all comparisons.

***Summarising judgments across the 6 domains***

The final output of CINeMA is a table with the level of concern for each of the 6 domains. We choose to summarise judgments across domains using the 4 levels of confidence of the GRADE approach: very low, low, moderate, or high:

1. If two or more domains were rated with “major concerns” or if one domain was rated with “major concerns” and one or more domains were rated “some concerns”, we downgraded the overall certainty of evidence to “very low”.

2. If no domains were rated with “major concerns” and three or more domains were rated with “some concerns”, we downgraded the overall certainty of evidence to “low”.

3. If no domains were rated with “major concerns” and two or less domains were rated with “some concerns”, we downgraded the overall certainty of evidence to “moderate”.

4. If all the domains were rated with “no concerns”, we kept the overall certainty of evidence to “high”.

Due to factors that may reduce the confidence in a treatment effect may affect more than 1 domain. Indirectness includes consideration of intransitivity, which is manifested as statistical incoherence in the data. Heterogeneity will increase the imprecision of treatment effect and may be related to the variability of bias within the study or the existence of reporting bias. In addition, in the presence of heterogeneity, the ability to detect important discontinuities will be reduced. Therefore, the 6 CINeMA domains should be considered jointly rather than in isolation to avoid downgrading the overall level of confidence more than once for related concerns.

**Reference**

1. Papakonstantinou, T., Nikolakopoulou, A., Higgins, J. P., Egger, M., & Salanti, G. (2020). Cinema: software for semiautomated assessment of the confidence in the results of network meta‐analysis. *Campbell systematic reviews*, *16*(1), e1080.

2. Ostinelli, E. G., Schulze, M., Zangani, C., Farhat, L. C., Tomlinson, A., Del Giovane, C., ... & Cortese, S. (2025). Comparative efficacy and acceptability of pharmacological, psychological, and neurostimulatory interventions for ADHD in adults: a systematic review and component network meta-analysis. *The Lancet Psychiatry*, *12*(1), 32-43.
